# Sex and gender aspects in diabetes mellitus: Focus on access to health care and cardiovascular outcomes

**DOI:** 10.3389/fpubh.2023.1090541

**Published:** 2023-02-02

**Authors:** Teresa Gisinger, Zahra Azizi, Pouria Alipour, Jürgen Harreiter, Valeria Raparelli, Karolina Kublickiene, Maria Trinidad Herrero, Colleen M. Norris, Khaled El Emam, Louise Pilote, Alexandra Kautzky-Willer

**Affiliations:** ^1^Gender Medicine Unit, Division of Endocrinology and Metabolism, Department of Internal Medicine III, Medical University of Vienna, Vienna, Austria; ^2^Centre for Outcomes Research and Evaluation, McGill University Health Centre Research Institute, Montreal, QC, Canada; ^3^Department of Translational Medicine, University of Ferrara, Ferrara, Italy; ^4^University Center for Studies on Gender Medicine, University of Ferrara, Ferrara, Italy; ^5^Faculty of Nursing, University of Alberta, Edmonton, AB, Canada; ^6^Section for Renal Medicine, Department of Clinical Science, Intervention, and Technology (CLINTEC), Karolinska Institute and Karolinska University Hospital, Stockholm, Sweden; ^7^Clinical and Experimental Neuroscience (NiCE-IMIB-IUIE), School of Medicine, University of Murcia, Murcia, Spain; ^8^Heart and Stroke Strategic Clinical Networks-Alberta Health Services, Toronto, ON, Canada; ^9^Electronic Health Information Laboratory, Children's Hospital of Eastern Ontario Research Institute, Ottawa, ON, Canada; ^10^Faculty of Medicine, University of Ottawa, Ottawa, ON, Canada; ^11^Replica Analytics Ltd, Ottawa, ON, Canada; ^12^Divisions of Clinical Epidemiology and General Internal Medicine, McGill University Health Centre Research Institute, Montreal, QC, Canada; ^13^Gender Institute La Pura, Gars am Kamp, Austria

**Keywords:** diabetes mellitus, sex differences, gender medicine, cardiovascular health, public health

## Abstract

**Aims:**

The aim of this study was to elucidate whether sex and gender factors influence access to health care and/or are associated with cardiovascular (CV) outcomes of individuals with diabetes mellitus (DM) across different countries.

**Methods:**

Using data from the Canadian Community Health Survey (8.4% of respondent reporting DM) and the European Health Interview Survey (7.3% of respondents reporting DM), were analyzed. Self-reported sex and a composite measure of socio-cultural gender was constructed (range: 0–1; higher score represent participants who reported more characteristics traditionally ascribed to women). For the purposes of analyses the Gender Inequality Index (GII) was used as a country level measure of institutionalized gender.

**Results:**

Canadian females with DM were more likely to undergo HbA1c monitoring compared to males (OR = 1.26, 95% CI: 1.01–1.58), while conversely in the European cohort females with DM were less likely to have their blood sugar measured compared to males (OR = 0.88, 95% CI: 0.79–0.99). A higher gender score in both cohorts was associated with less frequent diabetes monitoring. Additionally, independent of sex, higher gender scores were associated with higher prevalence of self-reported heart disease, stroke, and hospitalization in all countries albeit European countries with medium-high GII, conferred a higher risk of all outcomes and hospitalization rates than low GII countries.

**Conclusion:**

Regardless of sex, individuals with DM who reported characteristics typically ascribed to women and those living in countries with greater gender inequity for women exhibited poorer diabetes care and greater risk of CV outcomes and hospitalizations.

## 1. Introduction

The importance of the influence of sex and gendered factors on the risk of/and outcomes of non-communicable diseases is increasingly being recognized. Incorporation of gender dimensions in research has provided novel insights on how sex and gender influence the epidemiology, pathophysiology, treatment, and outcomes of various diseases. The investigation of sex differences includes biologically-linked diversity between males and females, which results from sex chromosomes, sex-specific gene expression of autosomes, sex hormones, and their effect on organ systems (1, 2). Gender however is a socio-cultural construct that differs between individuals and can be investigated by measuring gendered factors that are categorized with domains including gender identity, roles, relations, and institutionalized gender (3). The gender inequality index (GII), a proxy measure of institutionalized gender, was established in 2017 by the United Nations (UN) Health Reports. It measures the gender inequality in a nation based on reproductive health, empowerment and economic status (4). Country-specific differences in economic, health care and educational systems can lead to differences in gender equality with the resulting potential of impacting overall health status.

In the field of endocrinology and metabolism, sex, and gender differences in diabetes mellitus (DM) have been identified (5). Current literature describes sex differences in prevalence, symptoms, comorbidities, outcomes, treatments, and prevention of individuals with DM. For example, males with diabetes have a higher risk for microvascular complications, such as retinopathy and nephropathy (6), whereas several studies report a higher relative risk for cardiovascular diseases in females with diabetes compared to their male counterparts (7–9). This higher risk may be related not only to biological, but also to gendered environmental and behavioral factors (5, 10). Finally, the occurrence of type 2 diabetes mellitus at a younger age in females is associated with higher rates of cardiovascular outcomes, requiring more aggressive treatment strategies, including screening (11). The higher incidence of cardiovascular complications in females with diabetes mellitus could be explained by the fact that females who transition from normoglycemia to type 2 diabetes mellitus are more likely to have other cardiovascular risk factors compared to their male counterparts as inflammation, central obesity, hypercoagulability, dyslipidemia, hypertension, insulin resistance, and its associated endothelial dysfunction (12–14). Nevertheless it is reported that females are undertreated when suffering from diabetes mellitus (15). Still not only biological sex, but also psychosocial and socioeconomic status influences diabetes mellitus outcome. For example females are more likely to follow a healthy diet compared to males (16). However, males are more often physical active than females (16).

While research has amplified the importance of metabolic disorders in assessing cardiovascular risk, less is known about the effect of gender-related variables on the management and outcomes of individuals with diabetes. Therefore, our international transatlantic *Gender Outcomes INternational Group: to Further Wellbeing Development* (GOING-FWD) consortium investigated and compared the effect of gender-related factors on the management and clinical outcomes of individuals with diabetes mellitus in Europe and Canada. Further, we stratified the European analyses in relation to the country specific GII.

## 2. Subject, materials, and methods

### 2.1. Study design

Data from the Canadian Community Health Survey (CCHS, 2015–2016, *N* = 109,659, % female = 53.7) and the Second Wave of the European Health Interview Survey (E-HIS-second wave, 2013–2015, *N* = 316,333, %female = 51.3) were analyzed.

The EHIS is an European health information survey that is administered every 5 years (17). The first wave was conducted between 2006 and 2009, and a second wave was administered between 2013 and 2015 in all European Union member states, Iceland, and Norway. The countries included in the second wave were as follow; 2013: Belgium and the United Kingdom; 2014: Bulgaria, the Czech Republic, Estonia, Greece, Spain, France, Croatia, Italy, Cyprus, Latvia, Lithuania, Luxembourg, Hungary, Malta, Netherlands, Austria, Poland, Portugal, Romania, Slovenia, Slovakia, Finland, and Sweden; 2015: Denmark, Germany, Ireland, Iceland, and Norway.

The CCHS is a cross-sectional survey collecting information regarding physical and mental health status, social determinants of health, and health care resource utilization in the Canadian population (18). This survey has been administered every 2 years from 2001 to 2005, and then annually since 2007. The target subjects in this survey include individuals 12 years of age and above who live in an Canadian province (*n* = 10) or territory (*n* = 3). The exclusion criteria included those living on reserves, or aboriginal settlements, individual serving in the Canadian forces, institutionalized populations, children living in foster care (12–17 years), individuals living in Région du Nunavik and Région des Terres-Cries-de-la-Baie-James in the Quebec health territories. The questions from both surveys pertinent to the current analyses are reported in Appendices 1, 2. EHIS as well as CCHS collected information concerning health conditions (self-perceived health, chronic diseases, accidents,...), health care (usage of health care, usage of drugs, unmet needs), health-related determinants (physical activity, weight, fruit, and vegetable consumption,…), gendered-sociodemographic variables (sex, age, country of birth, nationality, income, education, employment status,…), and national health system metrics (health of children, more information on chronic diseases) (17, 18).

### 2.2. Going FWD methodology

This study is part of research conducted by members of the GOING-FWD Consortium, a five-country multidisciplinary project with the goal of integrating sex and gendered factors in the assessment of outcomes of chronic diseases. A multistep approach for identifying gender-related variables and outcomes in both surveys was utilized based on GOING-FWD methodology for retrospective studies (19–21). In order to compare databases data harmonization was taken place. First we used the GOING-FWD systematic multistep approach for retrospective studies in order to identify gender-related variables and outcomes in both databases (19). Then to identify gender-related factors, the Women Health Research Network's gender framework was used as well as the GOINGFWD wish list. This framework divides gender into: gender identity, gender roles, gender relationships, and institutionalized gender. In order to compare these two datasets, we performed a retrospective data harmonization according to the Maelström Research guidelines. Hence two datasets in which all variables of interest were categorized in the same way were created. Next we selected the datasets from 2015 in order to have maximum compatibility.

### 2.3. Study outcomes

In both surveys, outcome measures included (1) diabetes monitoring used as a proxy for health care access (reported as checking blood sugar in the past 12 months by a health professional in European countries and checking HbA1c in the past 12 months by a health professional in Canada), (2) the prevalence of cardiovascular diseases including heart disease or stroke, and (3) rates of hospitalizations.

In the EHIS survey the question and the available answers were as follows “*Last time of blood sugar measurement by a health professional*: Available answers—*1: Within the past 12 months, 2: 1–*<*3 years, 3: 3–*<*5 years, 4: More than 5 years, 5: Never*. While in the CCHS survey, the dichotomic (i.e., yes or no) question for measuring HbA1c in Canadians included*: “In the past 12 months, has a health care professional tested you for hemoglobin ‘A one-C'?*” (An “A one-C” hemoglobin test measures the average level of blood sugar over a 3-month period). In EHIS the presence of cardiovascular disease and stroke was asked as followed “*Suffering from coronary heart disease or angina pectoris in the past 12 months”* and “*Suffering from a stroke, cerebral hemorrhage, cerebral thrombosis in the past 12 months.”* The answer options were dichotomic (i.e., yes or no). In EHIS hospitalization rate was reported as a dichotomic question (i.e., yes or no) more specifically “*Admission as an inpatient in a hospital in the past 12 months*.” In CCHS the presence of cardiovascular disease and stroke was also a dichotomic question “*Do you have heart disease?”* and “*Do you suffer from the effects of a stroke?*.” Also in CCHS hospitalization rate was reported as a dichotomic question “*In the past 12 months, have you been a patient overnight in a hospital?”*

### 2.4. Gender inequality index

European countries were stratified based on their GII reported for the years of survey data collection (4) and were categorized into three categories gender inequality categories including low (GII < 0.077), medium (GII: 0.077–0.1635) and high (≥0.1635). A higher GII means higher gender inequality for women. Low GII countries included; Belgium, Denmark, Finland, Netherlands, Norway, Sweden, Slovenia; Medium GII Countries included Austria, Cyprus, Czech Republic, Croatia, Germany, Greece, France, Ireland, Iceland, Italy, Lithuania, Luxemburg, Poland, Portugal, Spain, and UK; and High GII Countries were Bulgaria, Estonia, Hungary, Malta, Latvia, Romania, and Slovakia. Canada has GII of 0.083 similar to medium GII countries in Europe.

### 2.5. Statistical analysis and gender score measure

Descriptive results were reported as mean and standard deviation for continuous variable and frequency and percentage for categorical variables. *T*-test and Chi-Square tests were utilized for comparing the baseline characteristics and gender-related variables between individuals with and without type 1 or type 2 diabetes mellitus, and males and females with type 1 or type 2 diabetes mellitus. Non-parametric tests were used for non-normally distributed data. To ensure the statistical power of the tests, the case analysis (pairwise deletion) approach was used to address missing data.

**Figure 1:**
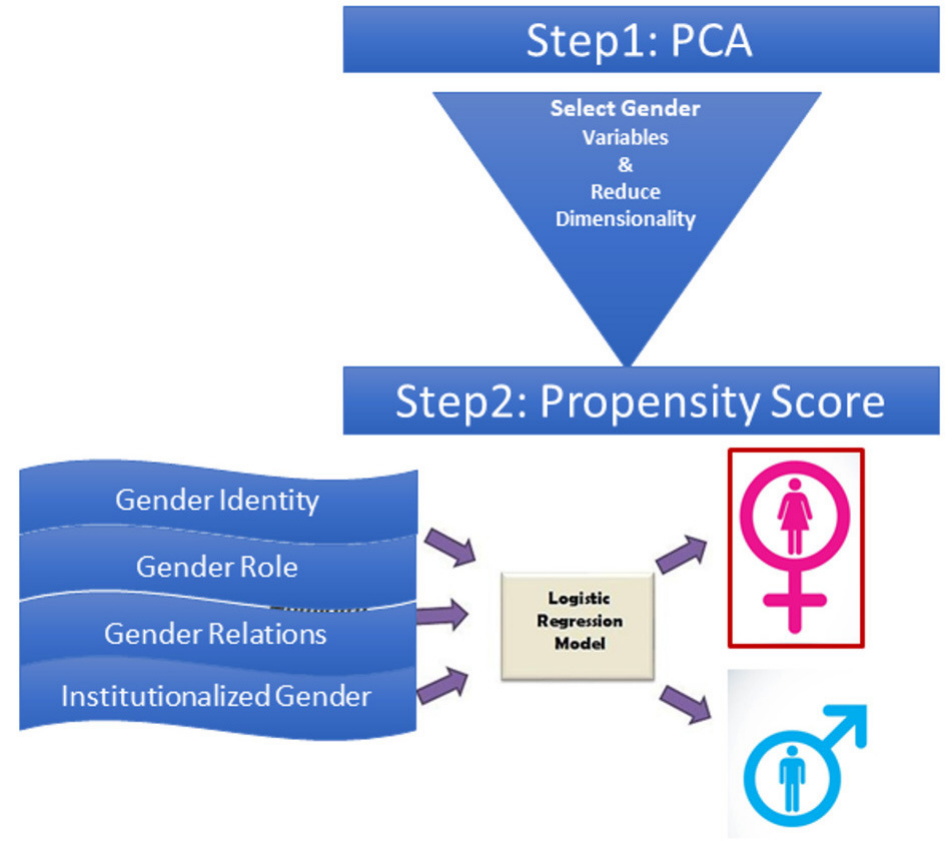
Gender score calculation method. Gender index was calculated through the construction of a propensity score, which was derived from coefficient estimates in the final logistic regression model with biological sex as dependent variable and gender related variables as covariates. The propensity score for each person was defined as the conditional probability of being a female vs. a male based on gender-related variables. This score ranges from 0 to 1, with higher scores relating to characteristics traditionally ascribed to women.

**Figure 2:**
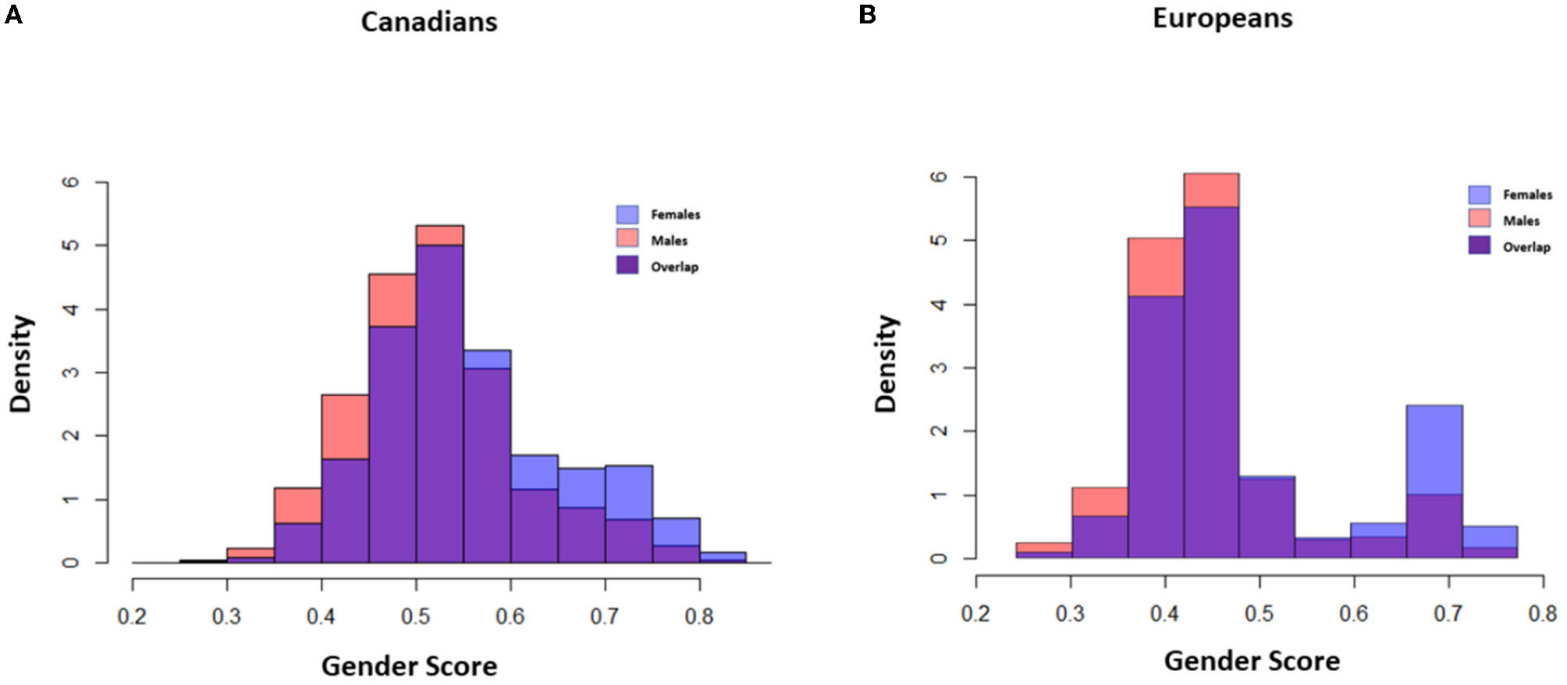
Gender score distribution in Canadians and Europeans. Density Plot: Y Axis: probability density of gender, X Axis: gender score I: Gender score distribution in male and females in Canadian **(A)** and European populations **(B)**. Red, Gender score in males; Blue, Gender score in females; Purple, Overlapping of Gender score in both groups. Higher gender score demonstrates characteristics traditionally ascribed to women in the population. The distribution of the gender score in men and women did not entirely overlap with biological sex in both populations which shows their partially independent effect. Moreover, the distribution was more polarized in Europeans and there was less overlap between roles in the society.

The relationship between gender-related variables and diabetes outcomes such as diabetes monitoring, reported outcomes (heart disease and stroke), and rates of hospitalizations were assessed using a logistic regression model adjusted for age, body mass index, and comorbidities. Gender-related variables were assessed individually and as a composite measure using a gender score. The GENESIS-PRAXY methodology was used to create a composite measure of gender from a selected set of gender-related variables using a principal component analysis (PCA) method and propensity score (22, 23). The detailed methodology has been reported previously (24). In brief, PCA was used to select gender-related variables from psychosocial variables extracted from both surveys (24). The PCA helps with reducing the dimensionality of data and the selection of variable sets with the most variation in the database. Components that accounted for >80% cumulative variance in the datasets were selected (Appendices 3, 4). Variables with factor loading >0.4 were selected from each component. Therefore, different country-specific components were used in the European and the Canadian databases. The gender score was constructed with a composite of household size (CCHS and EHIS), perceived life stress (CCHS), education level (EHIS and CCHS), sense of belonging to community (CCHS), marital status (EHIS and CCHS), and household income (EHIS and CCHS). The final set of gender-related variables was then used in a multivariable model with female sex as the dependent variable and gender-related variables as covariates (Figure 1). The gender score was created by calculating a propensity score based on the final logistic regression model. The propensity score is a measure of the conditional probability of being female based on the coefficients of gender-related variables in the model. Higher gender scores represent characteristics that are traditionally ascribed to women including greater stress level, being widowed or divorced, larger household size, more formal education in Canadians and lower level of education in Europeans, sense of belonging to community, and lower income (score range: 0–1) (Appendix 5, Figure 2).

Moreover, in European countries the models were adjusted for the country-specific GII. A 2-way sex-by-gender variable interaction was assessed in repeated sets of univariable/multivariable models between each gender variable and sex.

Finally, a sensitivity analysis was performed to assess the unique role of gender stratified by sex.

Data analysis was performed using R software (Version 4.0.3). *P* ≤ 0.05 were considered to be statistically significant.

## 3. Results

### 3.1. Study population characteristics

Amongst all participants in the EHIS (*N* = 316,333, Females: 51.3%) and CCHS (*N* = 109,659, Females: 53.7%), 7.3 and 8.4% reported having diabetes mellitus, respectively. Compared to participants without diabetes, those with diabetes had higher BMIs (EHIS: 28.9 vs. 25.6 kg/m^2^, *P* < 0.001; CCHS: 29.8 vs. 26.33 kg/m^2^, *P* < 0.001), the majority were older than 50 years (EHIS: 95.4 vs. 68.2%, *P* < 0.001; CCHS: 87.7 vs. 52.3%, *P* < 0.001) and more than half had hypertension (EHIS: 60.7 vs. 23.9%, *P* < 0.001; CCHS: 58.1 vs. 21.5%, *P* < 0.001).

A majority of the individuals with diabetes (EHIS: 83.8%, CCHS: 51.3%) had a lower level of formal education (i.e., at/or below secondary level). Moreover, more than 60% of the participants with diabetes in Canada and 59.7% in Europe were in the low to middle income group (Tables 1A, B).

**Table 1A:** Baseline characteristics of Canadian and European populations in the 2014–2016 surveys in overall and patients with diabetes.

	**CCHS**					**E-HIS**				

**Baseline characteristic, %**	**Overall**	**Diabetes** +			***P*** **-value** ^**^	**Overall**	**Diabetes** +			***P*** **-value** ^**^
		**Male**	**Female**	***P*** **-value** ^*^			**Male**	**Female**	***P*** **-value** ^*^	
	***N*** = **109,659**	***N*** = **4,782**	***N*** = **4,441**			***N*** = **316,333**	***N*** = **11,081**	***N*** = **12,021**		
**Age**										
< 20	10.1	0.6	0.7	< 0.001	< 0.001	5.4	0.4	0.5	< 0.001	< 0.001
20–29	10.7	1	1.3			11.5	1.2	1.7		
30–39	13.8	2.8	3.5			14.8	2.4	3.1		
40–49	13.1	7.5	7.3			17.5	7.1	6		
50–59	16.6	18.9	16.9			18	19.1	16.1		
60–69	17.8	32.7	28.1			17.1	32.9	29.7		
70–79	11.6	25.5	27.1			15.6	37.0	43.0		
≥80	6.3	11.1	15							
**Sex-female**										
	53.7	–	–		< 0.001		–	–	–	< 0.001
**BMI (mean, SD)**										
	26.33 (5.27)	29.78	29.8	0.7	< 0.001	25.85	28.82	29.06	< 0.001	< 0.001
**HX smoking**										
Never	40.3	23.6	39	< 0.001	< 0.001	75.5	79	87.1	< 0.001	< 0.001
Former	41.1	60.3	45.5			–	–	–		
Occasionally	4.5	3	2.8			4.5	2.9	1.9		
Daily	14.1	13	12.6			18.4	16.7	9.4		
**HX hypertension**										
	21.5	55.5	60.8	< 0.001	< 0.001	23.9	57.1	64	< 0.001	< 0.001
**HX diabetes**										
	8.4	–	–			7.3	–	–	–	–
**Alcohol intake**										
Never	24.7	29.2	43.2	< 0.001	< 0.001	29.7	29.4	58.9	< 0.001	< 0.001
< Once/month	25	25.3	34.6			30.3	22.1	27.9		
2–4 times/month	21.9	18.5	12.6			19.6	17.4	6.7		
2–3 times/week	15.8	13.1	5.7			8.3	9	2.2		
4–6 times/week	5.6	4.6	1.7			2.7	3.2	0.6		
Daily	7	9.3	2.3			9.4	18.9	3.7		

The data is presented as percentage of participants unless otherwise specified.

^*^P-value: The difference between male and female in diabetes patients.

^**^P-value: The difference between diabetes and non-diabetes patients.

+: People with diabetes mellitus.

**Table 1B:** Descriptive result of gender related variables in Canadian and European populations in the 2014–2016 surveys in overall and patients with diabetes.

	**CCHS**					**E-HIS**				

**Gender variables, %**	**Overall**	**Diabetes** +			***P-*** **value** ^**^	**Overall**	**Diabetes** +			***P-*** **value** ^**^
		**Male**	**Female**	***P*** **-value** ^*^			**Male**	**Female**	***P*** **-value** ^*^	
	***N*** = **109,659**	***N*** = **4,782**	***N*** = **4,441**			***N*** = **316,333**	***N*** = **11,081**	***N*** = **12,021**		
**Marital status**										
Single	29.1%	14.4%	13.2%	< 0.001	< 0.001	27.6%	11.5%	8.3%	< 0.001	< 0.001
Divorced/widowed	20%	21.2%	43.8%			17.8%	16.7%	44.1%		
Common-law/married	59.9%	64.4%	43%			54.6%	71.8%	47.6%		
**Household size**										
1	27.4%	30.5%	45.4%	< 0.001	< 0.001	18.5%	19.4%	34.9%	< 0.001	< 0.001
2	35.4%	52%	39.4%			33.9%	53.1%	40.6%		
3	13.7%	9.1%	8.2%			19.5%	14.3%	12%		
4	14.4%	5.3%	3.8%			17.8%	8.1%	6.8%		
5 & 5+	9.1%	2.9%	3.2%			10.3%	5.2%	5.6%		
**Education**										
< Secondary	23%	26.6%	31.5%	< 0.001	< 0.001	14.9%	27%	37.8%	< 0.001	< 0.001
Secondary	21.5%	22%	22.9%			56.5%	53.7%	48.9%		
Post-secondary or	55.6%	51.4%	45.7%			10.1%	6.6%	6%		
Grad	0%	0%	0%			18.6%	12.7%	7.3%		
**Worked last 12 months**										
	61.5%	44.3%	33.7%	< 0.001	< 0.001	47.4%	24.8%	15.4%	< 0.001	< 0.001
**Occupation type**										
Self employed	16.1%	25%	14.4%	< 0.001	< 0.001	15.6%	19.3%	14.4%	< 0.001	< 0.001
Employee	83.9%	75%	85.6%			84.4%	80.7%	85.6%		
**Working hours**				
Part-time	82%	15.5%	27.6%	< 0.001	< 0.001	16.5%	9.9%	29.1%	< 0.001	0.033
Full-time		84.5%	72.4%			83.5%	90.1%	70.9%		
**Household Income**										
Low	9.8%	11.2%	20%	< 0.001	< 0.001	39.4%	36.5%	52.6%	< 0.001	< 0.001
Medium	35.3%	43.9%	47.8%			20.3%	20.1%	21.9%		
High	54.8%	44.9%	32.2%			40.3%	43.5%	25.5%		
**Absence from work (due to health-related conditions)**										
	1.2%	11.1%	14.8%	0.3	0.07	33%	29.9%	45.2%	< 0.001	< 0.001
**Absence from work (due to taking care of someone)**										
	0.2%	0%	4.8%	0.005	< 0.001	–	–	–		
**Having child (**<**15 years)**										
	–	–	–	–		23.4%	9.2%	9.8%	0.126	< 0.001
**Having child (**<**12 years)**										
	19.7%	5.5%	6.5%	0.03	< 0.001	–	–	–	–	–
**Immigrant**										
	15.7%	17.2%	16.7%	0.4	< 0.001	8.5%	8%	8.2%	–	0.056
**Racial group**										
White	88.1%	89.8%	90.4%	0.4	< 0.001	–	–	–	–	–
Other	11.9%	10.2%	9.6%							
**Perceived life stress: stress during the day**										
Not at all	15.3%	23.8%	18.2%	< 0.001	< 0.001	–	–	–	–	–
Not very	25.6%	25.2%	24.2%							
A bit	39.6%	34%	36.2%							
Quite a bit	16.6%	13.5%	17.3%							
Extremely	2.9%	3.6%	4.1%							
**Sense of belonging to community**										
Very weak	7.2%	8.2%	9.5%	< 0.001	< 0.001	–	–	–	–	–
Somewhat weak	22%	21.3%	20.5%							
Somewhat strong	50.4%	47.8%	46%							
Very strong	20.4%	22.7%	24%							
**House ownership**										
Landlord	73.6%	74.4%	65.1	< 0.001	< 0.001	–	–	–	–	–
Tenant	26.4%	25.6%	34.9							
**Parent with child in the house**										
	23.2%	13.3%	13.6%	0.7	< 0.001	33.8%	52.5%	36.6%	< 0.001	< 0.001

^*^P-value: The difference between male and female in diabetes patients.

^**^P-value: The difference between diabetes and non-diabetes patients.

+: People with diabetes mellitus.

The mean gender score was 0.54 ± 0.09 in both populations. Individuals with diabetes had higher gender scores compared to the population without diabetes (EHIS: 0.57 ± 0.10, *P* < 0.001; CCHS: 0.56 ± 0.10, *P* < 0.001). The distribution of the gender index in both cohorts revealed a more polarized distribution in European countries, with less overlap between gender roles when compared to the Canadian cohort (Figure 2).

### 3.2. Health care access among individuals with diabetes

Overall, the 85% of Canadians reported having their HbA1c levels monitored by a health care professional in the past year. Similarly, nearly 92.4% of individuals with diabetes in Europe had their blood sugar measured by a healthcare professional in the past year. The self-reported prevalence of heart disease (19.1 vs. 17.2%,) and stroke (5.3 vs. 5.1%) were similar, while the rate of all cause overnight hospitalization in the past 12 months was 14.5 and 22.2% in CCHS and EHIS, respectively.

### 3.3. Role of sex and gender variables in diabetes care

**Figure 3:**
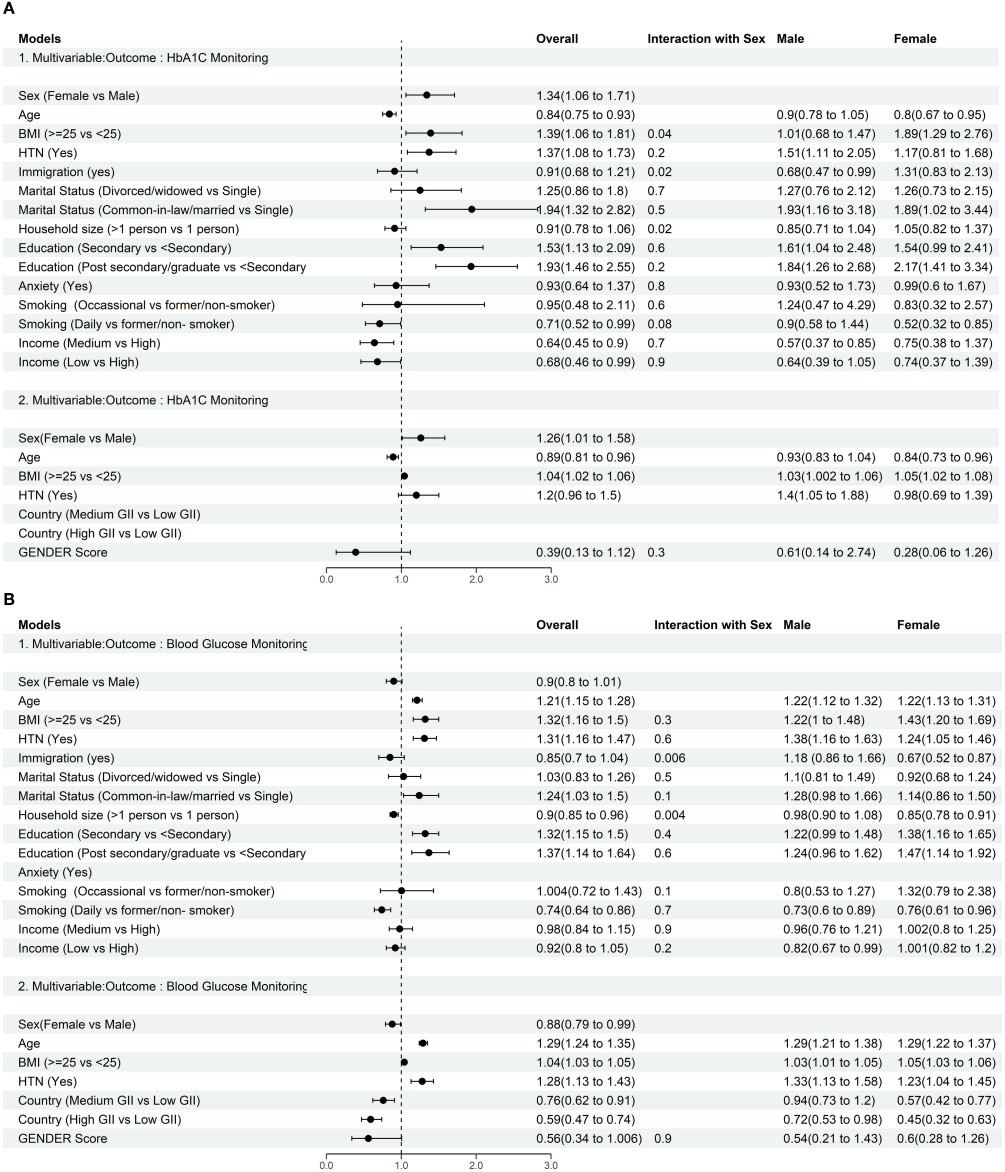
**(A)** Forest plot: Assessing role of biological sex and gender variables in care of individuals with diabetes including rate of HbA1c monitoring by health care professional in the past 12 months in Canadian population: Results are presented as Odds Ratio (95% CI). Interaction between sex and gender was assessed via repeated sets of multivariable models including two-way interaction between each gender variable and sex. **(B)** Forest plot: Assessing role of biological sex and gender variables in care of individuals with diabetes including prevalence of blood glucose monitoring by health care professional in the past 12 months in European population: Results are presented as Odds Ratio (95% CI). Low GII Countries: GII < 0.077: Belgium, Denmark, Finland, Netherlands, Norway, Sweden, Slovenia; Medium GII Countries: GII: 0.077–0.1635: Austria, Cyprus, Czech Republic, Germany, Greece, France, Spain, Croatia, Ireland, Iceland, Italy, Luxemburg, Poland, Portugal, UK, Lithuania; High GII Countries: GII > 0.1635: Bulgaria, Estonia, Hungary, Malta, Romania, Slovakia, Latvia. Interaction between sex and gender was assessed via repeated sets of multivariable models including two-way interaction between each gender variable and sex.

Biological sex was a significant determinant of diabetes care monitoring in individuals with diabetes. Compared to males, females in Canada were more likely to undergo diabetes care monitoring (OR = 1.26, 95% CI: 1.01–1.58) (Figure 3A, Appendix 6), while females in Europe were less likely to undergo diabetes care monitoring compared to males (OR: 0.88, 95% CI: 0.79–0.99) (Figure 3B, Appendix 7).

The results of the multivariable analyses (Figure 3A) to assess diabetes care revealed that a higher BMI (≥25 kg/m^2^), hypertension, being married, and a higher level of formal education were associated with greater likelihood of diabetes monitoring in all countries. Moreover, being a daily smoker, was associated with a lower likelihood of diabetes monitoring regardless of country. A significant difference between the Canadian and European cohorts was observed in the roles of age, household size and income on diabetes monitoring. Older age in Canadians was associated with a reduced likelihood of HbA1c monitoring (OR = 0.84, 95% CI: 0.75–0.93), while in Europeans it was associated with a higher likelihood of glucose monitoring (OR = 1.21, 95% CI: 1.15–1.28). Among gender-related variables, only a larger household size in Europe (OR = 0.9, 95%CI: 0.85–0.96) and a lower income in Canada (OR = 0.68, 95% CI: 0.46–0.99) were significantly associated with lower likelihood of diabetes care (Figures 3A, B, Appendices 6, 7).

### 3.4. Sex-stratified models for diabetes monitoring

In the sex-stratified female models, higher BMI (CCHS, OR = 1.89, 95% CI: 1.29–2.76; EHIS, OR = 1.43, 95% CI: 1.20–1.69), and higher educational attainment (CCHS: OR = 2.17, 95% CI: 1.41–3.34; EHIS: OR = 1.47, 95% CI: 1.14–1.92) were associated with greater likelihood of diabetes care in both female cohorts. Being married was associated with better care uniquely in females in the Canadian cohort (as measured by HbA1c) whereas having hypertension and older age was associated with better care in the European cohort. While, being an immigrant (daily smoker) and a larger household size, and being married were associated with a lower likelihood of blood glucose check in Europeans, being older age and daily smoker were associated with less likelihood of HbA1c monitoring in Canadian female participants (Figures 3A, B, Appendices 6, 7).

When assessing factors associated with higher likelihood of diabetes care amongst males, different results were obtained. Having concomitant hypertension was significantly associated with diabetes care for both cohorts. Additionally, a higher formal education level, and being married were associated with a higher likelihood of diabetes monitoring in the Canadian male cohort; in the European cohort, older age, and greater BMI were strongly associated with diabetes monitoring.

Males with lower income had worse diabetes care regardless of the country cohort, whereas being an immigrant in Canada and daily smoker in Europe were associated with less likelihood of HbA1c or glucose monitoring (Figures 3A, B, Appendices 6, 7).

### 3.5. Role of sex and gender variables in cardiovascular outcomes and hospitalizations of diabetes

In the multivariable models used for assessing the role of sex and gender-related variables in CV outcomes of individuals with diabetes, female sex was associated with a lower risk of heart disease (CCHS OR = 0.54, 95% CI: 0.47–0.61; EHIS OR = 0.8, 95% CI:0.73–0.87), and stroke (CCHS OR = 0.6, 95% CI: 0.48–0.77; EHIS OR = 0.76, 95% CI: 0.65–0.88) (Appendices 8–11, 14–17).

Older age, hypertension, being widowed or divorced, and lower income were associated with an increased prevalence of self-reported heart disease and stroke, and rate of hospitalizations in both the overall and sex-stratified models regardless of country (Appendices 8–19).

Anxiety was associated with a greater risk of heart disease, stroke, and hospitalization only in the Canadian cohort. In sex-specific models anxiety was only significant in predicting outcomes among females. Additionally, a larger household size was associated with a significantly higher risk of heart disease and stroke only in European females (Appendices 8–19).

### 3.6. The association between the composite gender score and outcomes

Independent of sex, higher gender scores (representing more characteristics typically ascribed to women), were associated with lower diabetes monitoring (CCHS: OR = 0.39, 95% CI: 0.13–1.12; EHIS: OR = 0.56, 95% CI: 0.34–1.006) in both cohorts. Moreover, Europeans with diabetes were less likely to check their blood glucose levels in countries with higher GII (more gender inequality toward women) compared to countries with low GII. These results were also consistent across the sex-stratified models (Figures 3A, B, Appendices 6, 7).

Additionally, a higher gender score, independent of sex, was associated with increased risk of hospitalization (CCHS OR = 6.14, 95% CI: 3.29–11.46; EHIS OR = 1.92, 95% CI: 1.33–2.77), and higher prevalence of self-reported heart disease (CCHS OR = 5.63, 95% CI: 3.17–10.001; EHIS OR = 2.63, 95% CI: 1.75–3.95) and stroke (CCHS OR = 13.08, 95% CI: 4.64–36.97; EHIS OR = 1.96, 95% CI: 0.96–3.97). In Europe, countries with medium-high GII had a higher risk of outcomes than low GII countries (Appendices 8–19).

## 4. Discussion

The main findings of the study are that female sex was associated with better diabetes monitoring in the Canadian population and clinical outcomes including prevalence of heart disease and stroke as well as the rate of hospitalizations in individuals with diabetes, whereas gendered factors traditionally ascribed to women were associated with worse outcome in both the European and Canadian cohorts. Furthermore, when investigating European countries with different levels of gender inequality, we observed less diabetes monitoring and a higher risk for adverse outcomes in participants living in countries with higher gender inequality.

While some studies have shown associations between gender-related factors and outcomes, the influence of gender-related factors on diabetes mellitus risk and adverse clinical outcome has not been extensively investigated. And while known gender-related variables as less formal education and a reported lower socioeconomic status were associated with a higher risk of diabetes mellitus and mortality, especially in women (25–28), our analysis demonstrated a higher odds ratio for diabetes outcomes in participants with gendered characteristics more typically ascribed to women.

Importantly we also identified that female sex was associated with a lower risk of hospitalization, stroke and heart disease but found gendered characteristics commonly ascribed to women were associated with a higher risk for hospitalization, stroke and heart disease. Hence, worse outcome of diabetes mellitus in female individuals might more likely be related to gendered factors as opposed to sex (biological) factors. Similarly, a study in Germany reported that low socioeconomic status (SES), which is often related to characteristics more typically ascribed to women, was inversely correlated to BMI, waist circumference and low physical activity, especially in females and that female individuals with low SES had a higher rate of undiagnosed diabetes mellitus and this effect was reduced in females with higher income (29).

Females with diabetes have been observed to have a higher metabolic and cardiovascular risk compared to males, and it has been hypothesized that female individuals need to be exposed to a higher metabolic insult in order to be confirmed as having diabetes (15, 30, 31). Males with diabetes mellitus are reported to be more likely to have a lower BMI than females (32, 33), and females with diabetes tend to be older, report less formal education, lower income, more comorbidities and more depression than males (34). A recent study suggested that an extended period of rotating night shift work (a non-biological factor) is associated with a modestly increased risk of type 2 diabetes in female individuals, which appears to be partly mediated through increase in body weight (35).

Studies have showed that unpaid housework and responsibilities in the family may contribute to feelings of conflicting demands and sustained stress levels in females, even in matched highly educated group of employees (36, 37). It is recognized that the imbalance between the ability to adapt to environmental demands and overexposure to environmental stress, increases the risk of cardiometabolic diseases *via* neuroendocrine, autonomic and immune mediators (38). This may be partially explain our results where individuals with reported higher levels of stress that resulted in higher gender scores also had worse outcomes related to diabetes.

Even though there are numerous differences in culture and healthcare systems within Europe and Canada, this study obtained similar results concerning the influence of sex and gender on diabetes outcome. Interestingly though, we identified a higher effect of gender on diabetes outcome in the European cohort.

Of note, our study used the GII to investigate the country-related effect of gender inequality on diabetes monitoring and outcomes. The GII for Canadian cohort indicates that as a country Canada has lower level of gender inequality compared to the countries in the European cohort. This may have contributed to the finding of the differences in the gender scores between the two cohorts. After categorizing the European countries into three by GII groupings, we demonstrated that the effect gender on diabetes was more pronounced in countries with higher gender inequality. This may partially explain why the effect of gender on diabetes outcomes was higher in the overall European cohort with varying individual country GIIs, compared to the Canadian GII. Further, the higher magnitude of gender impact on diabetes among countries with higher GII supports the hypothesis that gender-related factors do play a relevant role in shaping the outcome of diabetes. In countries with high gender inequality, females may have more difficulties getting access to healthcare and/or report higher levels of stress due to lower incomes and greater household responsibilities.

Our findings should be interpreted in light of several limitations. The first is that we used self-reported data. Indeed, the usage of self-reported data could lead to over- and underestimated frequency of diseases and frequency of medical care usage. The second is that the EHIS did not differentiate between type 1 and type 2 diabetes. Due to a worldwide higher prevalence of diabetes mellitus type 2, the authors anticipated that the majority of this cohort would have consisted of individuals with type 2 diabetes mellitus (39, 40). Finally, this was a cross-sectional design and not all the “diabetes outcomes” can be attributed to diabetes itself.

The novelty of the study is that we were able to demonstrate that over and above biological sex, gender-related variables may in fact play a greater role in the outcomes of individuals with diabetes mellitus. Therefore, in order to start to improve the outcomes of care and treatment for diabetes it is imperative that the treatment plan include the consideration of psycho-socio-cultural gendered factors.

In summary, independent of biological sex, individuals with diabetes and with gendered characteristics typically ascribed to women and those living in countries with greater gender inequality exhibited a poorer diabetes monitoring, a greater risk of cardiovascular outcomes, and higher hospitalization rates. Therefore, country-specific gender-related factors and gender inequality must be targeted for improving the health status and access to care of patients with diabetes mellitus.

## Data availability statement

The data analyzed in this study is subject to the following licenses/restrictions: Restrictions apply to the availability of some, or all data generated or analyzed during this study because they were used under license. The corresponding authors will on request detail the restrictions and any conditions under which access to some data may be provided. Requests to access these datasets should be directed to Eurostat.

## Author contributions

Data curation was done by TG and ZA. Conceptualization, funding acquisition, investigation, methodology, project administration, resources, software, supervision, validation, and visualization was conceived by TG, ZA, LP, and AK-W. TG did the formal analysis and wrote the initial draft of the manuscript. All authors contributed substantially to the discussion, reviewing and editing, and approved the final manuscript.
