# Supplementary material for: Sex and gender aspects in diabetes mellitus: Focus on access to health care and cardiovascular outcomes
Source: Front Public Health. 2023 Feb 2;11:1090541. doi: 10.3389/fpubh.2023.1090541 (PMC9932273; doi:10.3389/fpubh.2023.1090541)
Supplement: Supplementary file 1 [file Data_Sheet_1.docx]

**Appendix:**

**Appendix 1: E-HIS survey questions**

| **E-HIS Survey** | |
| --- | --- |
| Sex of respondent | Male/Female |
| Age of respondent in completed years at the time of the interview | Digit number |
| Country of birth | -Native-born  -Born in another EU Member State  -Born in non-EU country |
| Country of citizenship at time of data collection | -National/has citizenship of the reporting country  -Non-national/does not have citizenship of the reporting country but national of other EU Member States  -Non-national/does not have citizenship of the reporting country but non-EU country nationality |
| Legal marital status | -Never married and never been in a registered partnership  -Married or in a registered partnership  -Widowed or in registered partnership that ended with death of partner (not remarried or in new registered partnership)  -Divorced or in registered partnership that was legally dissolved (not remarried or in new registered partnership) |
| Number of persons living in household, including the respondent | Digit number |
| Number of persons aged younger as 4  Number of persons aged from 5 to 13 | Digit number |
| Highest level of education completed (Educational attainment) | -Based on ISCED-2011 classification -Early childhood development, pre-primary education -Primary education  -Lower secondary education  -Upper secondary education  -Post-secondary but non-tertiary education  -Tertiary education; short-cycle  -Tertiary education; bachelor level or equivalent -Tertiary education; master level or equivalent  -Tertiary education; doctoral level or  equivalent |
| Self-declared labour status | -Carries out a job or profession, including unpaid work for a family business or holding, an apprenticeship or paid traineeship, etc.  -Unemployed  -Pupil, student, further training, unpaid work experience  -In retirement or early retirement or has given up business  -Permanently disabled  -In compulsory military or community service  -Fulfilling domestic tasks  -Other inactive person |
| Full or part-time work | Full-time  Part-time |
| Status in employment | -Self-employed  -Employee with a permanent job/work contract of unlimited duration  -Employee with a temporary job/work contract of limited duration |
| Self-perceived general health: how a person perceives his/her health in general | Very good  Good  Fair  Bad  Very bad |
| Height (metres)/Weight (kilograms) |  |
| Type of smoking behaviour | Daily smoking  Occasional smoking  No smoking |
| Frequency of consumption of an alcoholic drink of any kind (beer, wine, cider, spirits, cocktails, premixes, liqueurs, homemade alcohol...) in the past 12 months | Every day or almost  5–6 days a week  3–4 days a week  1–2 days a week  2–3 days in a month  Once a month  Less than once a month  Not in the past 12 months, as I no longer drink alcohol  Never, or only a few sips or tries, in my whole life |
| Net monthly equivalised income of the household | Below 1st quintile Between 1st quintile and 2nd quintile 3 Between 2nd quintile and 3rd quintile 4 Between 3rd quintile and 4th quintile 5 Between 4th quintile and 5th quintile |
| Suffering from a stroke (cerebral haemorrhage, cerebral thrombosis) in the past 12 months | Yes  No |
| Suffering from a coronary heart disease or angina pectoris in the past 12 months | Yes  No |
| Suffering from high blood pressure in the past 12 months | Yes  No |
| Suffering from diabetes in the past 12 months | Yes  No |
| Difficulty in seeing, even when wearing glasses or contact lenses | No difficulty  Some difficulty  A lot of difficulty  Cannot do at all/Unable to do |
| Suffering from kidney problems in the past 12 months | Yes  No |
| Type of household | One-person household  Lone parent with child(ren) aged less than 25  Couple without child(ren) aged less than 25  Couple with child(ren) aged less than 25  Couple or lone parent with child(ren) aged less than 25 and other persons living in household  Other type of household |
| Last time of blood sugar measurement by a health professional | Within the past 12 months  1 to less than 3 years  3 to less than 5 years  More than 5 years  Never |
| Admission as an inpatient in a hospital in the past 12 months | Yes  No |

**Appendix 2: CCHS survey questions**

| **CANADIAN HEALTH SURVEY (CCHS)2014-16** | |
| --- | --- |
| Enter the respondent's sex. If necessary, ask: Is respondent male or female? | Male/ Female |
| What is your age? | 12 To 14 Years  15 To 17 Years  18 To 19 Years  20 To 24 Years  25 To 29 Years  30 To 34 Years  35 To 39 Years  40 To 44 Years  45 To 49 Years  50 To 54 Years  55 To 59 Years  60 To 64 Years  65 To 69 Years  70 To 74 Years  75 To 79 Years  80 Years Or More |
| Country of birth - Canada/other | Canada  Other |
| Immigrant | Landed immigrant / non-permanent resident Non-immigrant (Canadian born) |
| What is your marital status? Are you married, living common-law, widowed, separated, divorced, or single, never married? | Married  Common-Law  Widow/Sep/Div  Single/Never Mar |
| Household size | 1 Person  2 Persons  3 Persons  4 Persons  Grouped - 5 or more persons live in the household |
| Number of persons less than 12 years old in household | None  1 or More |
| Highest level of education – household, 3 levels | Less than secondary school graduation  Secondary school graduation, no post-secondary education  Post-secondary certificate diploma or univ degree |
| Highest level of education - respondent, 3 levels | Less than secondary school graduation  Secondary school graduation, no post-secondary education  Post-secondary certificate diploma or univ degree |
| Have you worked at a job or business at any time in the past 12 months? | Yes  No |
| Full-time / part-time working status | Full-Time  Part-Time |
| Were you an employee or self-employed? | Employee  Self-Employed |
| Perceived Health: In general, would you say your health is... ? | Excellent  Very good  Good  Fair  Poor |
| Perceived Mental Health: In general, would you say your mental health is...? | Excellent  Very good  Good  Fair  Poor |
| How would you describe your sense of belonging to your local community? Would you say it is...? | Very Strong  Somewhat Strong  Somewhat Weak  Very Weak |
| Thinking about the amount of stress in your life, would you say that most days are…? | Not at all stressful  Not very stressful  A bit stressful  Quite a bit stressful  Extremely stressful |
| Height (metres)/Weight (kilograms) |  |
| Type of smoker | Daily  Occasional  Not At All |
| During the past 12 months, how often did you drink alcoholic beverages? | < Once A Month  Once A Month  2 To 3 Times/Mo  Once/Week  2 To 3 Times/Wk  4 To 6 Times/Wk  Every Day |
| Total personal income from all sources | No Income  Less Than 20,000  $20,000-$39,999  $40,000-$59,999  $60,000-$79,999  $80,000 Or More |
| Total household income from all sources | No Or <$20,000  $20,000-$39,999  $40,000-$59,999  $60,000-$79,999  $80,000 Or More |
| Do you suffer from the effects of a stroke? | Yes  No |
| Do you have heart disease? | Yes  No |
| Remember, we’re interested in conditions diagnosed by a health professional and are expected to last or have already lasted 6  months or more. Do you have high blood pressure? | Yes  No |
| (Remember, we’re interested in conditions diagnosed by a health professional and that are expected to last or have already  lasted 6 months or more.)  Do you have diabetes? | Yes  No |
| Cultural / racial background | White  Non-white (Aboriginal or Other Visible Minority) |
| Is this dwelling… ? | Owned by member of hhld, even if it is still being paid for  Rented, even if no cash rent is paid |
| Living arrangement of selected respondent - (D, G) | Unattached individual living alone.  Unattached individual living with others.  Individual living with spouse/partner.  Parent living with spouse/partner and child(ren).  Single parent living with children.  Child living with a single parent with or without siblings.  Child living with two parents with or without siblings  Other |
| Tested for haemoglobin "A1C" - 12 mo : In the past 12 months, has a health care professional tested you for haemoglobin "Aone- C"? (An "A-one-C" haemoglobin test measures the average level of blood sugar over a 3-month period.) | Yes  No |
| Overnight hospital patient - 12 mo: [Excluding the time you spent in an emergency department] in the past 12 months, have you been a patient overnight in a hospital? | Yes  No |

**Appendix3. Factor loadings from the principal component analysis related to each gender variable in the selected components of CCHS database 2014-16**

| **PCA Analysis: CCHS** | **Component 1** | **Component 2** | **Component 3** | **Component 4** | **Component 5** | **Component 6** |
| --- | --- | --- | --- | --- | --- | --- |
| **Marital status** | 0.33 |  | **0.79** | 0.28 | 0.33 | 0.17 |
| **Household size** | **0.86** |  | -0.37 |  |  |  |
| **Education** |  | 0.12 | 0.28 | 0.10 | **-0.91** |  |
| **Working last 12 months** |  |  |  |  |  |  |
| **Working hours** |  |  |  |  |  |  |
| **Occupation category** |  |  |  |  |  |  |
| **Household Income** | -0.14 |  | -0.16 |  |  | **0.77** |
| **Racial group** |  |  |  |  |  | 0.18 |
| **Having children less than 12 years in family** | 0.20 |  |  |  |  | 0.21 |
| **Perceived life stress** |  | **0.95** |  | -0.26 | 0.1 |  |
| **Sense of belonging to community** |  | -0.24 | 0.30 | **-0.91** |  |  |
| **House ownership** |  |  | -0.10 |  |  |  |
| **Immigration** |  |  |  |  |  | 0.22 |
| **Being parent with children** | 0.23 |  |  |  |  | 0.23 |
| **Factor loadings from PCA analysis of gender related variables.**  **Cut off for Factor loading => 0.4** | | | | | | |

*Principal Component Analysis (PCA) methodology was used to reduce dimensionality and facilitate data compression to select the unique set of covariates to use in the predictive model. Ultimately, components that accounted for a cumulative variance of greater than 60% of the data were selected. Factor loadings (correlation between original variables and factors) with values of 0.4 and more were used to select the best set of variables. The optimized set of gender-related variables from the selected components in the PCA (supplement 1 and 2) were then used to create a multivariable logistic model with biological sex as the dependent variable and gender-derived components as covariates.*

**Appendix4: Factor loadings from the principal component analysis related to each gender variable in the selected components of E-HIS database**

| **PCA Analysis: E-HIS** | **Component 1** | **Component 2** | **Component 3** |
| --- | --- | --- | --- |
| **Marital status** | 0.366 | 0.177 | **0.806** |
| **Education** |  | **-0.806** | 0.371 |
| **Employment status** | **1.000** |  |  |
| **Working hours** |  |  |  |
| **Occupation category** |  |  |  |
| **Absent from work due to health problems** |  |  |  |
| **Income** | 0.150 | **-0.553** | -0.342 |
| **Having children less than 18 years in family** |  |  |  |
| **Immigration** |  |  |  |
| **Household Size** | **0.913** | 0.104 | -0.305 |
| **Perceived General health** |  |  |  |
| **Factor loadings from PCA analysis of gender related variables.**  **Cut off for Factor loading => 0.4** | | | |

*Principal Component Analysis (PCA) methodology was used to reduce dimensionality and facilitate data compression to select the unique set of covariates to use in the predictive model. Ultimately, components that accounted for a cumulative variance of greater than 60% of the data were selected. Factor loadings (correlation between original variables and factors) with values of 0.4 and more were used to select the best set of variables. The optimized set of gender-related variables from the selected components in the PCA (supplement 1 and 2) were then used to create a multivariable logistic model with biological sex as the dependent variable and gender-derived components as covariates.*

**Appendix5: Multivariable logistic models for assessing association of gender variables with biological sex as dependent variable for constructing gender score**

| **CCHS** | | **E-HIS** | |
| --- | --- | --- | --- |
| **Gender Variables** | **OR (95%CI)** | **Gender Variables** | **OR (95%CI)** |
| **Household size**  **(Reference:1Person)**  2 Persons  3 Persons  4 Persons  5 & 5+ Persons  **Perceived life stress:**  **stress during the day**  (Reference: 1=not at all)  2=not very  3=a bit  4=quite a bit  5=Extremely  **Education**  (Reference: <Secondary)  Secondary  Post secondary  **Sense of belonging to community**  (Reference:1=very weak)  2=somewhat weak  3=somewhat strong  4=very strong  **Marital Status**  (Reference: Single)  Divorced/widowed  Common-in-law/married  **Household Income**  (Reference: High)  Medium  Low | 1.46(1.40- 1.53)  1.49(1.42- 1.57)  1.56(1.48- 1.64)  1.67(1.58- 1.77)  1.35(1.30- 1.41)  1.55(1.49- 1.61)  1.79(1.71- 1.87)  1.52(1.40- 1.65)  1.13(1.09- 1.18)  1.17(1.13- 1.21)  1.06(1.01- 1.12)  1.16(1.11- 1.23)  1.20(1.13- 1.26)  2.69(2.58- 2.80)  1.07(1.03- 1.11)  1.30(1.26- 1.34)  1.71(1.63- 1.80) | **Household size**  **(Reference:1Person)**  2 Persons  3 Persons  4 Persons  5 & 5+ Persons  **Education**  (Reference: <Secondary)  Secondary  Post secondary  >Post secondary  **Marital Status**  (Reference: Single)  Divorced/widowed  Common-in-law/married  **Household Income**  (Reference: High)  Medium  Low | 1.17(0.13-0.18)  1.20(0.16-0.21)  1.22(0.17-0.23)  1.19(0.14-0.20)  1.10(0.07-0.12)  0.79(0.25-0.21)  0.88(0.16-0.10)  2.90(1.04-1.09)  1.12(0.09-0.13)  1.18(0.15-0.19)  1.32(0.26-0.29) |
| *Gender score was calculated using variables selected from PCA model in a multivariable model with sex as dependent variable and gender variables as covariates. The propensity score from coefficient estimates in the final logistic regression model which demonstrate the conditional probability of being a female versus a male based on gender-related variables constituted the gender score (ranging from 0-1). Higher gender score demonstrates characteristics traditionally ascribed to women.* | | | |

**Appendix6: Forest plot: Univariable model for assessing role of biological sex and gender variables in care of individuals with diabetes including rate of HbA1c monitoring by health care professional in the past 12 months in Canadian population:**

**Results are presented as Odds Ratio (95%CI)*

*§ Interaction between sex and gender was assessed via repeated sets of multivariable models including two-way interaction between each gender variable and sex.*


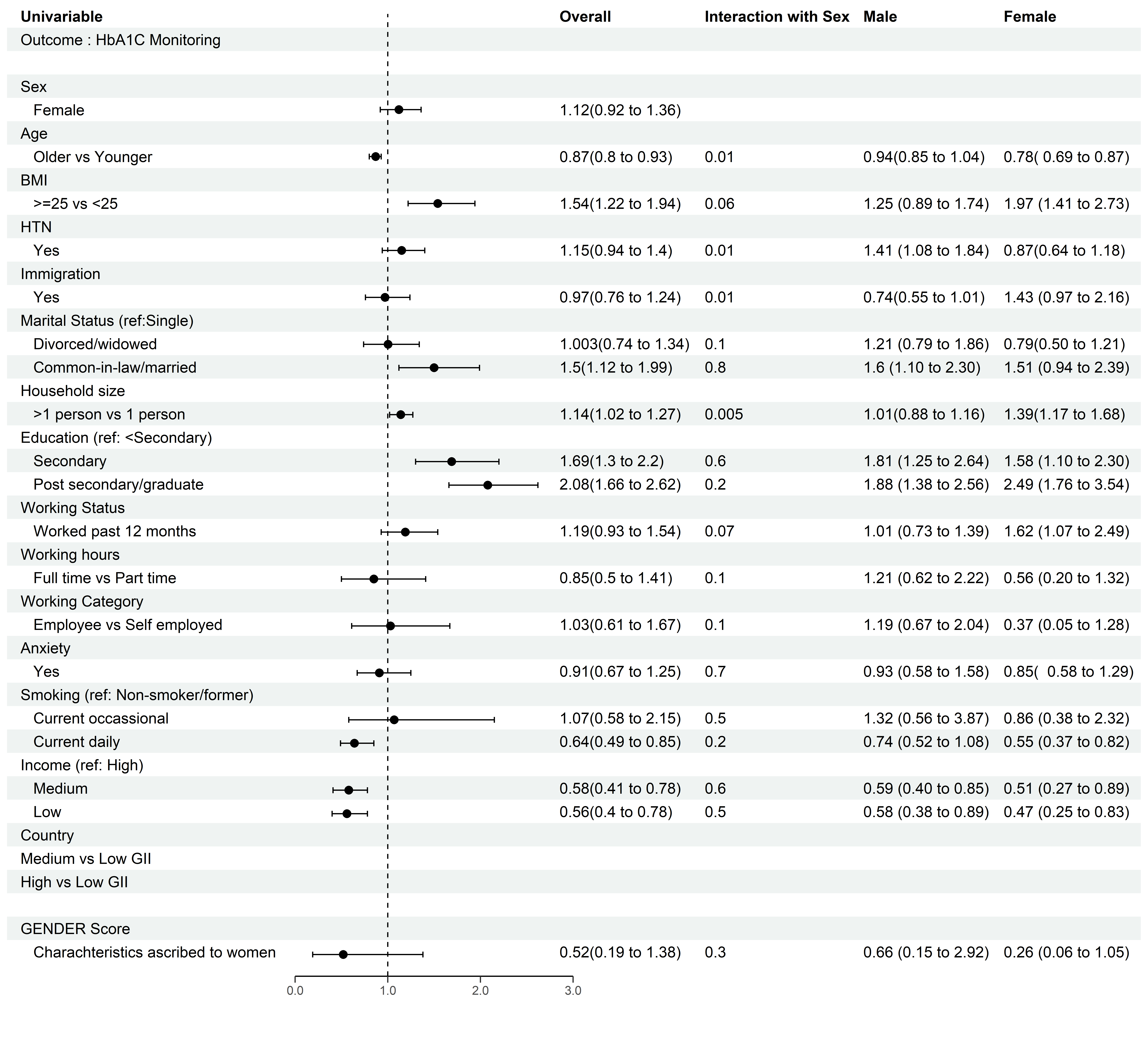


**Appendix7: Forest plot: Univariable model for assessing role of biological sex and gender variables in care of individuals with diabetes including prevalence of blood glucose monitoring by health care professional in the past 12 months in European population:**

**Results are presented as Odds Ratio (95%CI)*

*# Low GII Countries: GII <0.077: Belgium, Denmark, Finland, Netherlands, Norway, Sweden, Slovenia*

*Medium GII Countries: GII: 0.077-0.1635: Austria, Cyprus, Czech Republic, Germany, Greece, France, Spain, Croatia, Ireland, Iceland, Italy, Luxemburg, Poland, Portugal, UK, Lithuania;*

*High GII Countries: GII>0.1635: Bulgaria, Estonia, Hungary, Malta, Romania, Slovakia, Latvia*

*§ Interaction between sex and gender was assessed via repeated sets of multivariable models including two-way interaction between each gender variable and sex.*

*
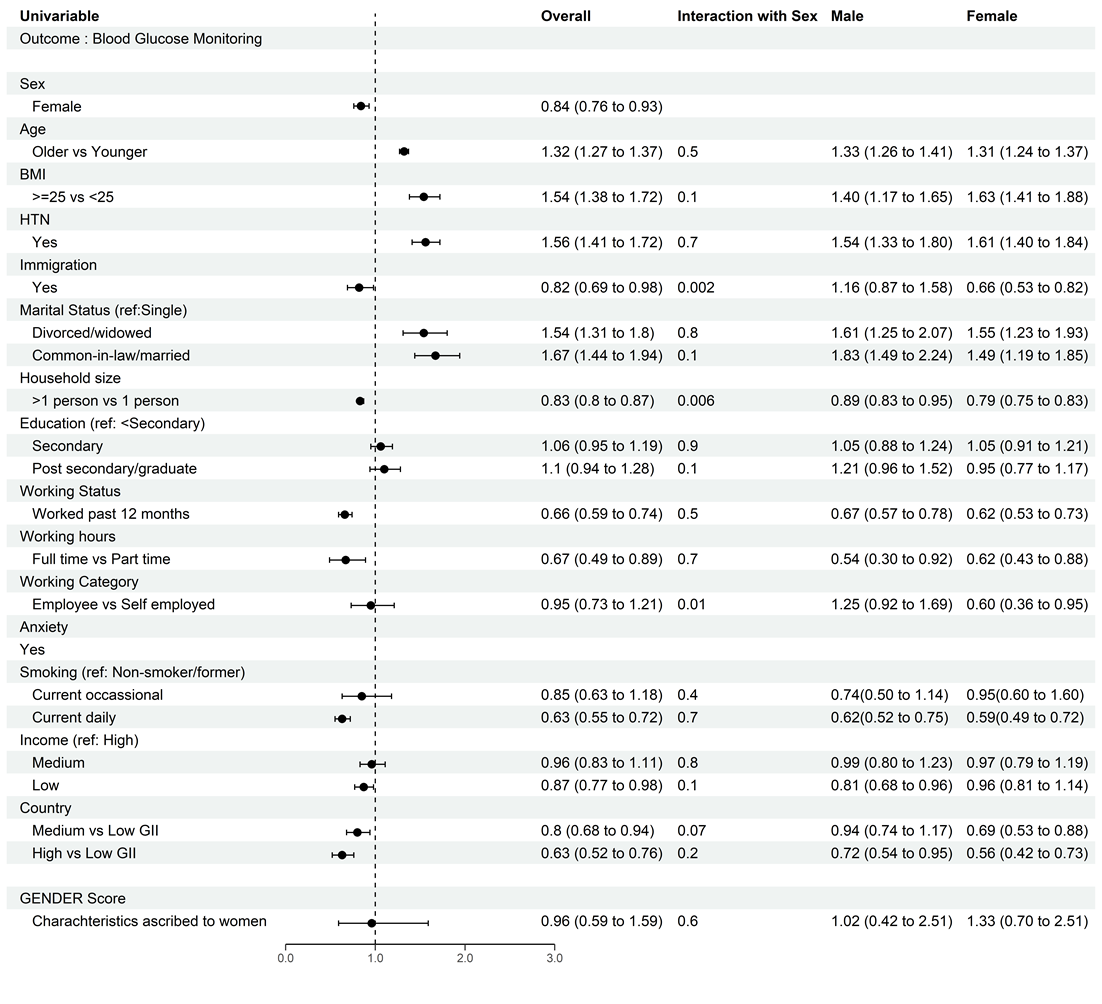
*

**Appendix8: Forest plot: Univariable model for assessing role of biological sex and gender variables in predicting heart disease of individuals with diabetes in Canadian population:**

**Results are presented as Odds Ratio (95%CI)*

*§ Interaction between sex and gender was assessed via repeated sets of multivariable models including two-way interaction between each gender variable and sex.*


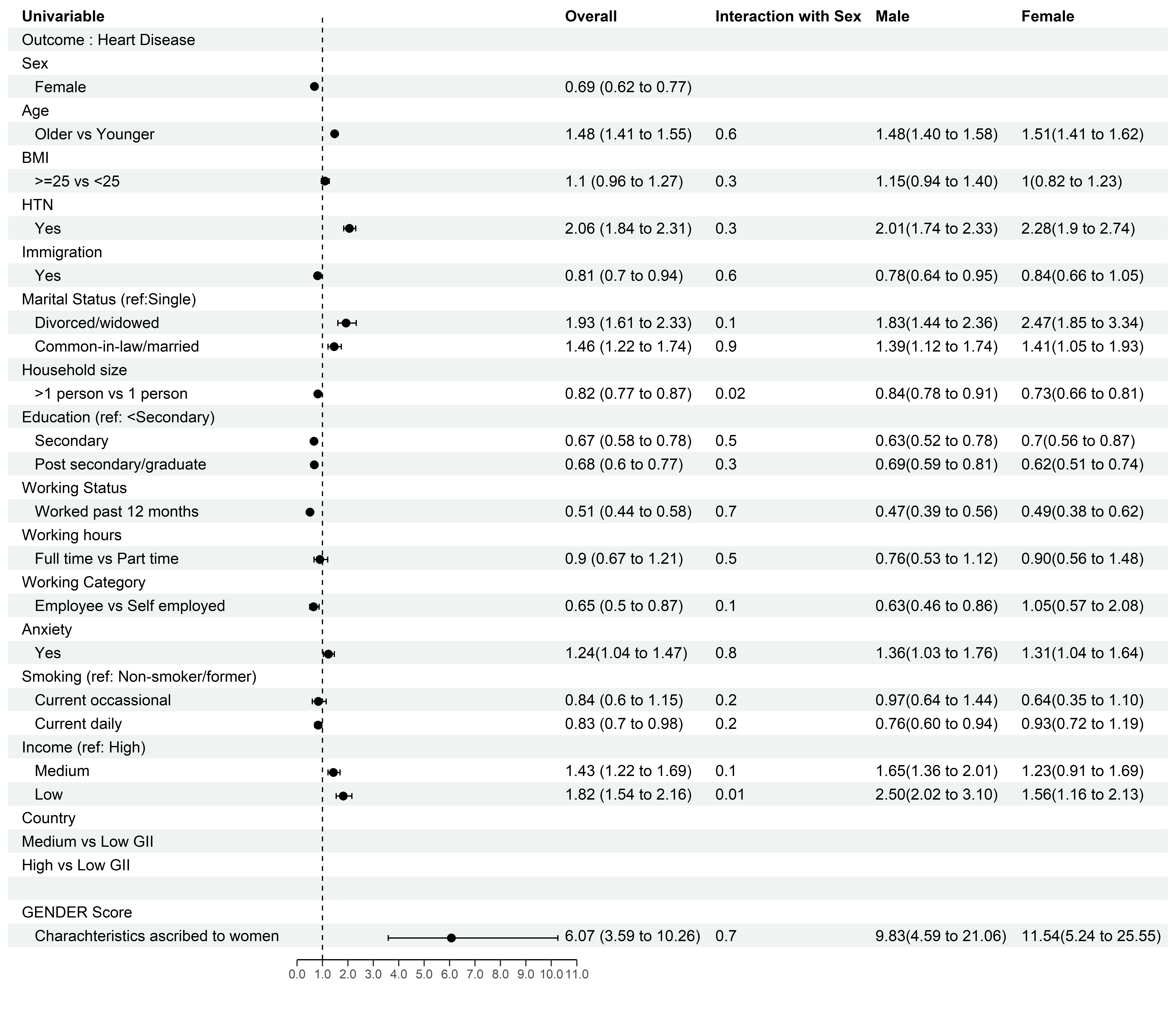


**Appendix9: Forest plot: Univariable model for assessing role of biological sex and gender variables in predicting heart disease of individuals with diabetes in European population:**

**Results are presented as Odds Ratio (95%CI)*

*# Low GII Countries: GII <0.077: Belgium, Denmark, Finland, Netherlands, Norway, Sweden, Slovenia*

*Medium GII Countries: GII: 0.077-0.1635: Austria, Cyprus, Czech Republic, Germany, Greece, France, Spain, Croatia, Ireland, Iceland, Italy, Luxemburg, Poland, Portugal, UK, Lithuania;*

*High GII Countries: GII>0.1635: Bulgaria, Estonia, Hungary, Malta, Romania, Slovakia, Latvia*

*§ Interaction between sex and gender was assessed via repeated sets of multivariable models including two-way interaction between each gender variable and sex.*


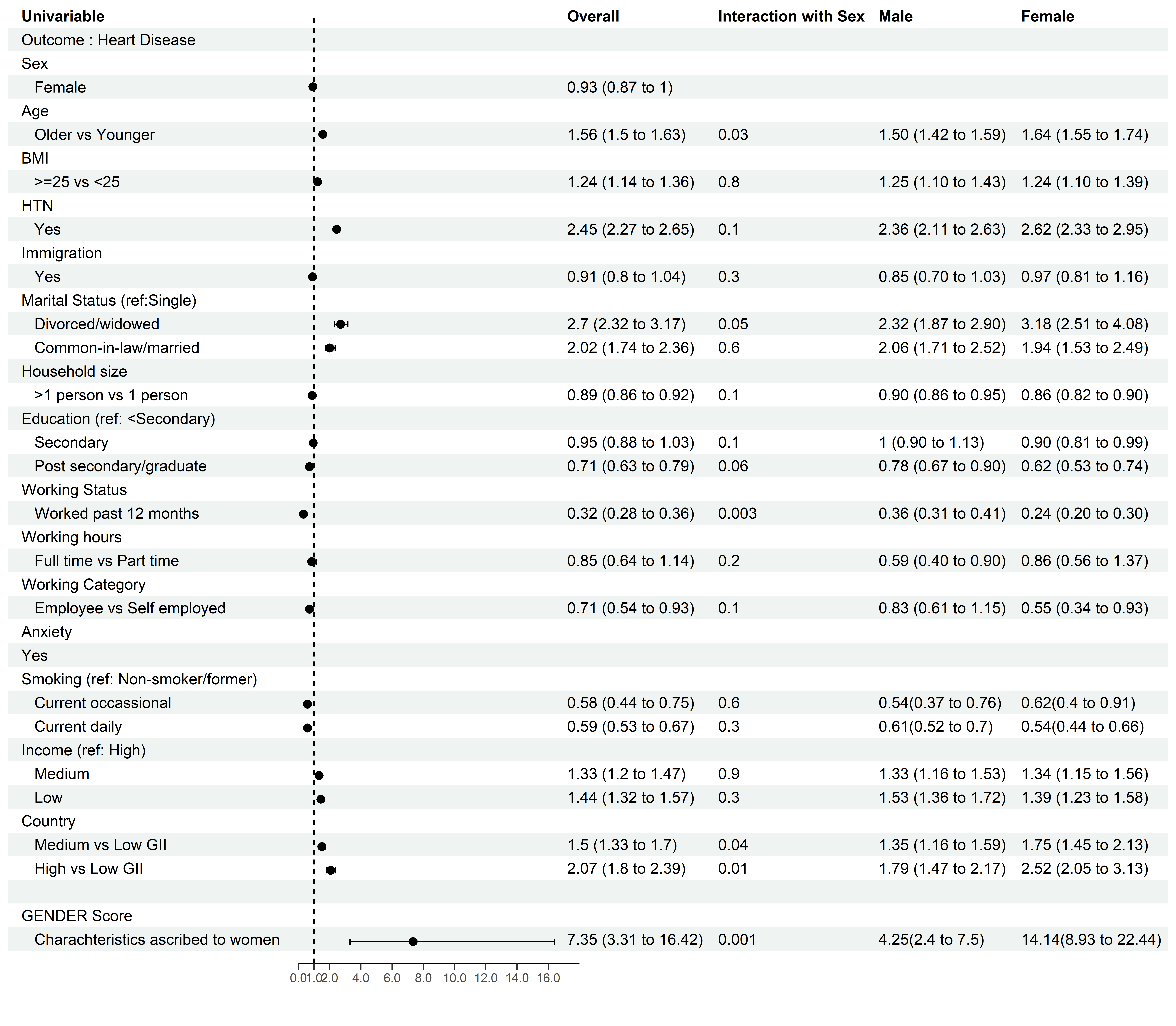


**Appendix10: Forest plot: Univariable model for assessing role of biological sex and gender variables in predicting stroke of individuals with diabetes in Canadian population:**

**Results are presented as Odds Ratio (95%CI)*

*§ Interaction between sex and gender was assessed via repeated sets of multivariable models including two-way interaction between each gender variable and sex.*


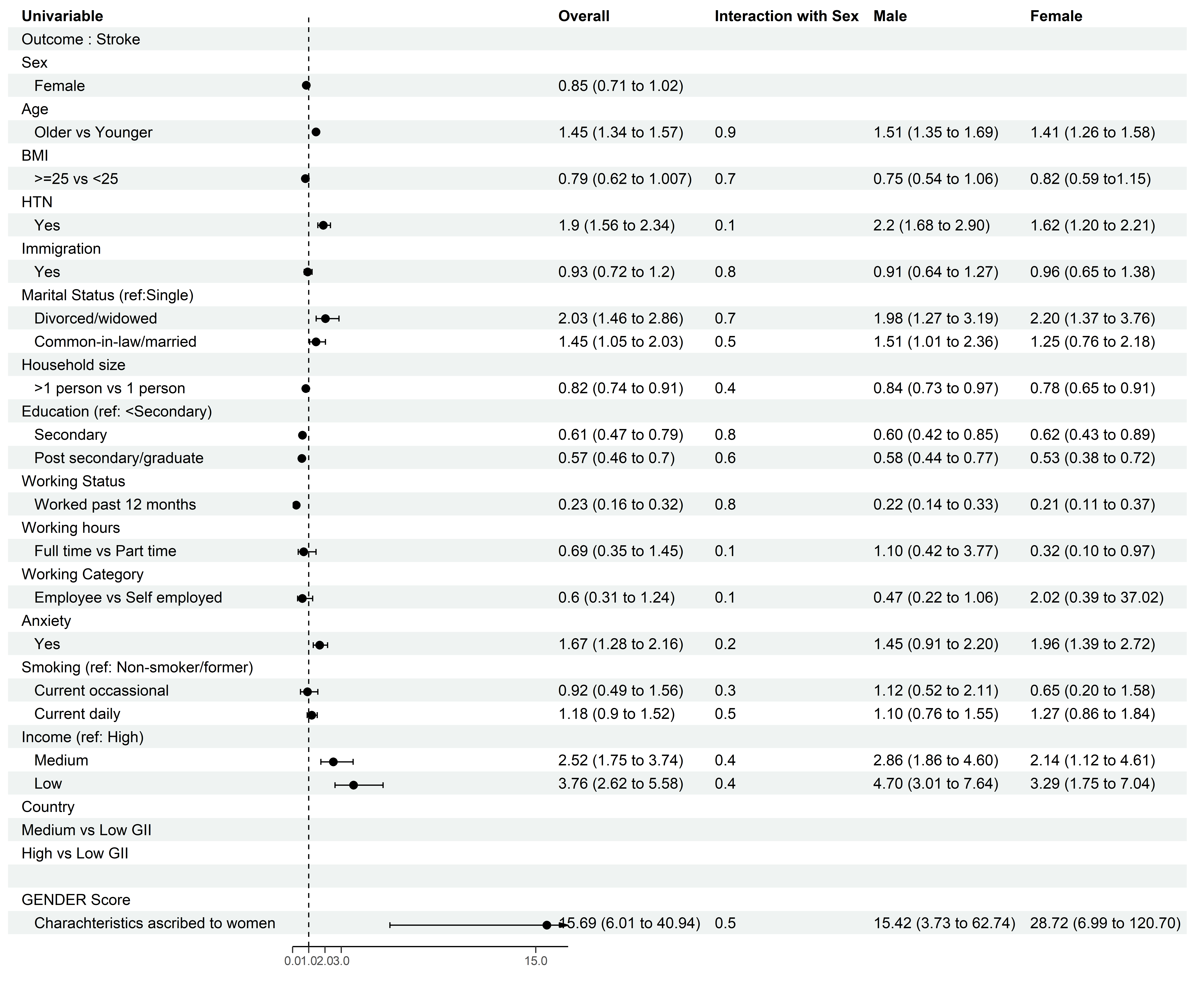


**Appendix11: Forest plot: Univariable model for assessing role of biological sex and gender variables in predicting stroke of individuals with diabetes in European population:**

**Results are presented as Odds Ratio (95%CI)*

*# Low GII Countries: GII <0.077: Belgium, Denmark, Finland, Netherlands, Norway, Sweden, Slovenia*

*Medium GII Countries: GII: 0.077-0.1635: Austria, Cyprus, Czech Republic, Germany, Greece, France, Spain, Croatia, Ireland, Iceland, Italy, Luxemburg, Poland, Portugal, UK, Lithuania;*

*High GII Countries: GII>0.1635: Bulgaria, Estonia, Hungary, Malta, Romania, Slovakia, Latvia*

*§ Interaction between sex and gender was assessed via repeated sets of multivariable models including two-way interaction between each gender variable and sex.*


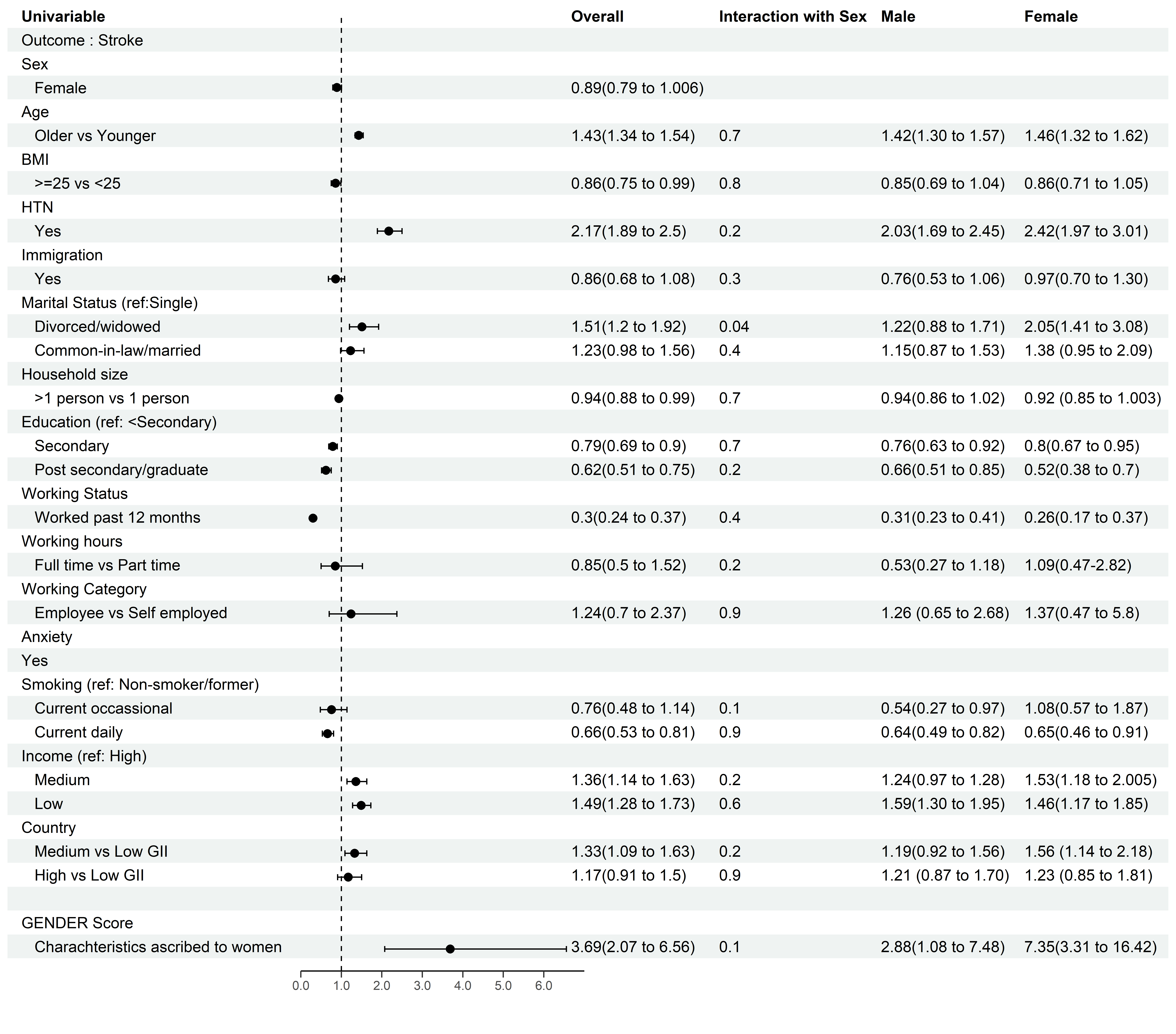


**Appendix12: Forest plot: Univariable model for assessing role of biological sex and gender variables in hospitalization rate of individuals with diabetes in Canadian population:**

**Results are presented as Odds Ratio (95%CI)*

*§ Interaction between sex and gender was assessed via repeated sets of multivariable models including two-way interaction between each gender variable and sex.*


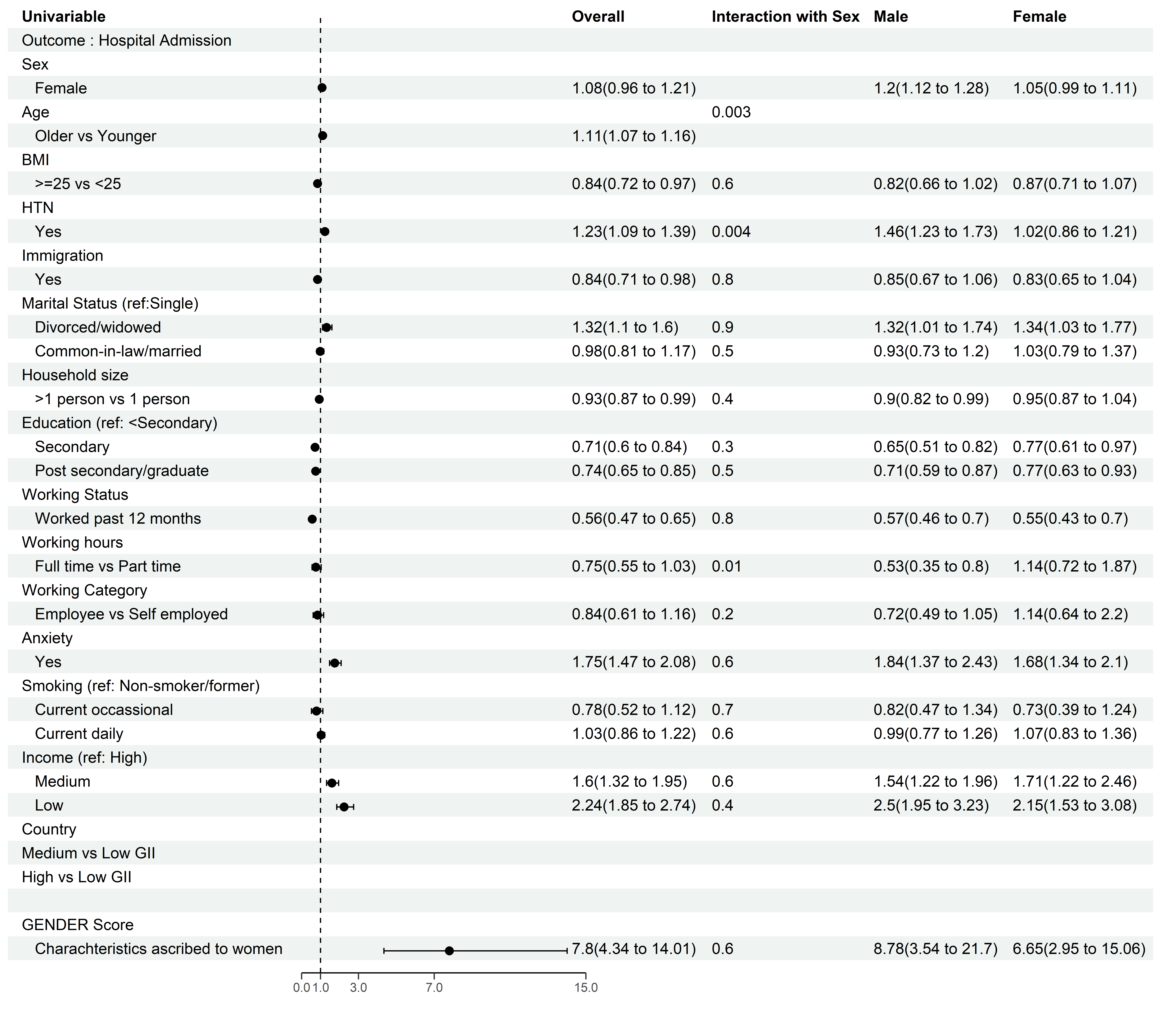


**Appendix13: Forest plot: Univariable model for assessing role of biological sex and gender variables in hospitalization rate of individuals with diabetes in European population:**

**Results are presented as Odds Ratio (95%CI)*

*# Low GII Countries: GII <0.077: Belgium, Denmark, Finland, Netherlands, Norway, Sweden, Slovenia*

*Medium GII Countries: GII: 0.077-0.1635: Austria, Cyprus, Czech Republic, Germany, Greece, France, Spain, Croatia, Ireland, Iceland, Italy, Luxemburg, Poland, Portugal, UK, Lithuania;*

*High GII Countries: GII>0.1635: Bulgaria, Estonia, Hungary, Malta, Romania, Slovakia, Latvia*

*§ Interaction between sex and gender was assessed via repeated sets of multivariable models including two-way interaction between each gender variable and sex.*


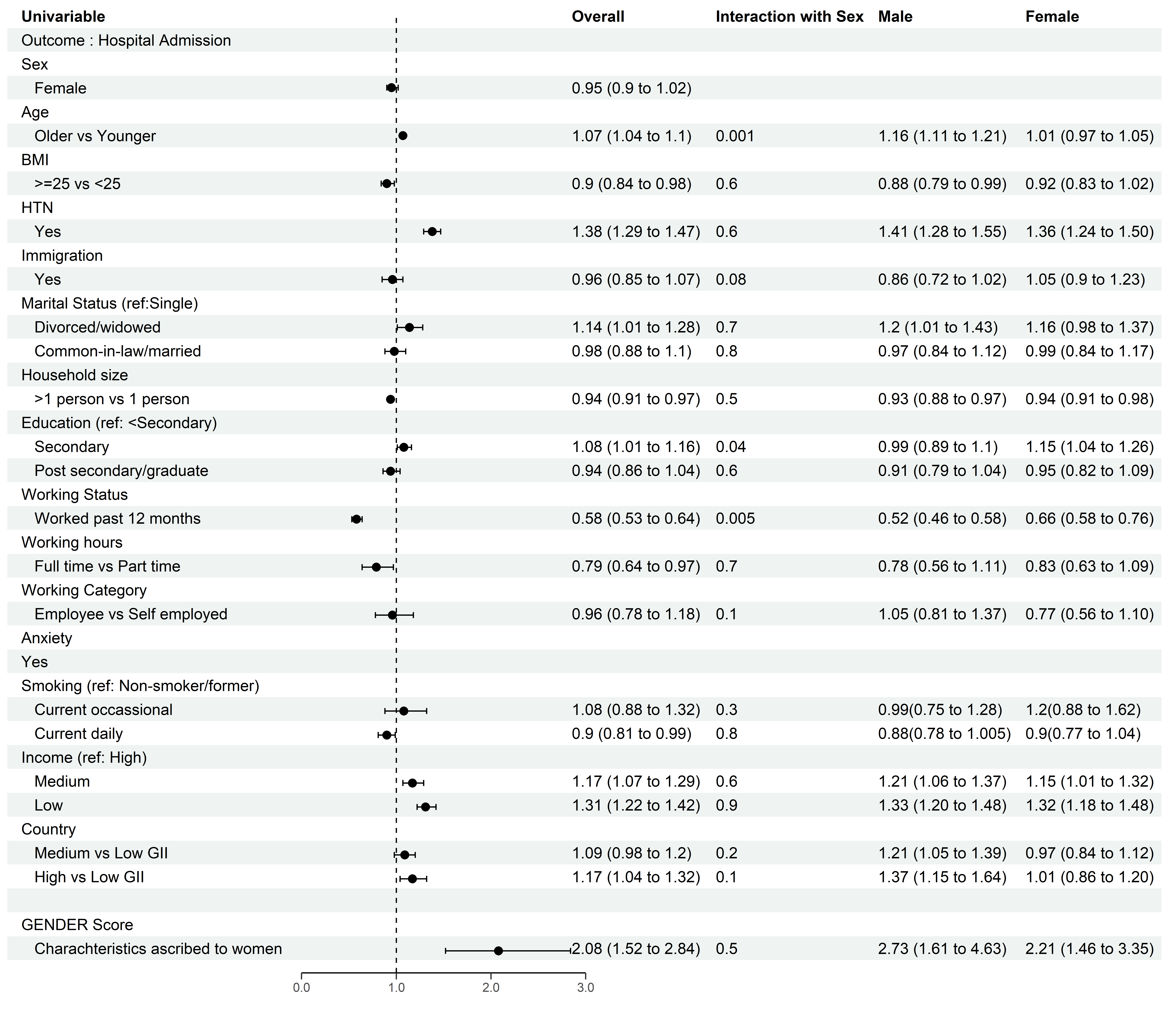


**Appendix14: Forest plot: Role of biological sex and gender variables in predicting heart disease of individuals with diabetes in Canadian population:**

**Results are presented as Odds Ratio (95%CI)*

*§ Interaction between sex and gender was assessed via repeated sets of multivariable models including two-way interaction between each gender variable and sex.*


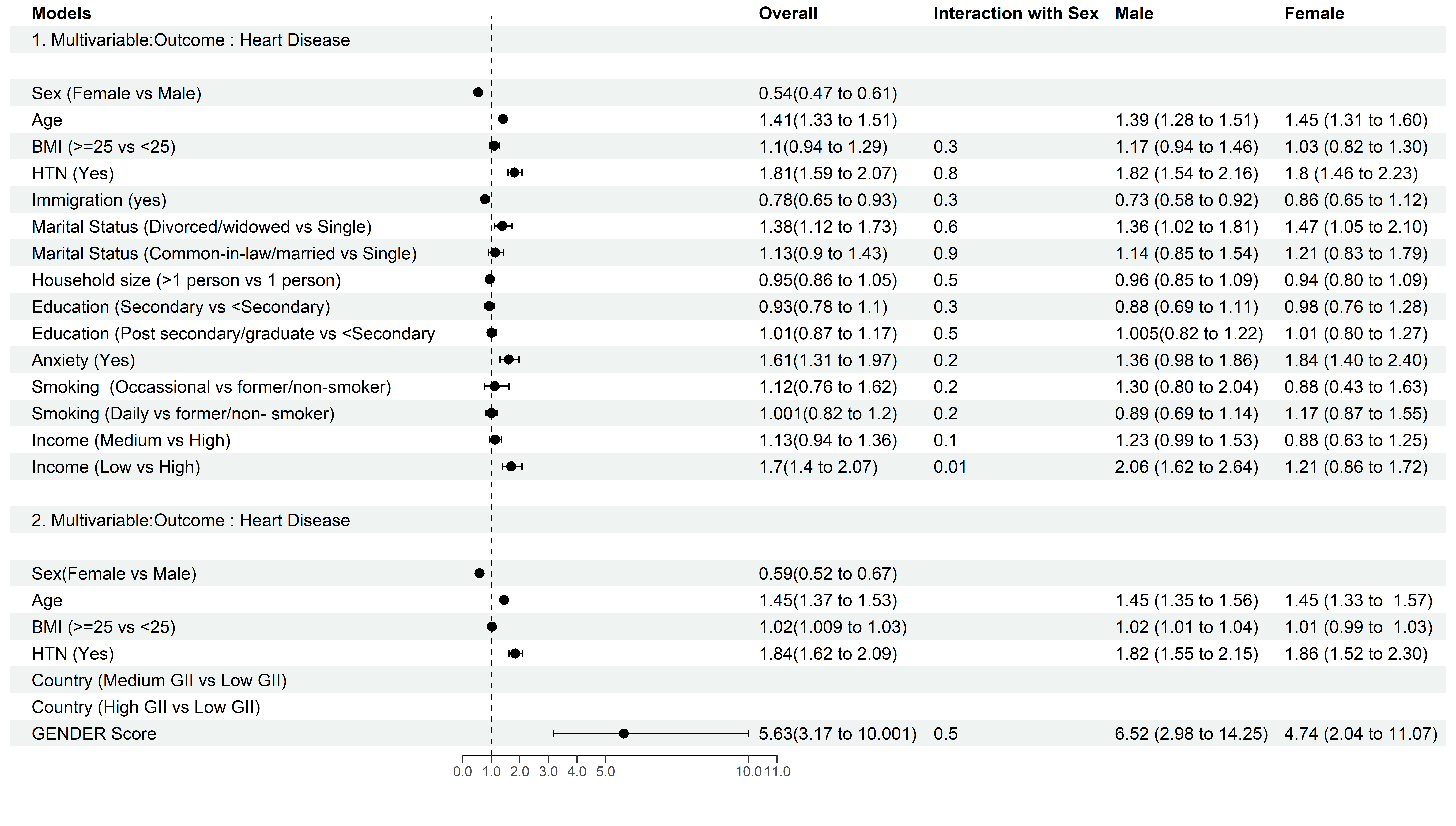


**Appendix15: Forest plot: Role of biological sex and gender variables in predicting heart disease of individuals with diabetes in European population:**

**Results are presented as Odds Ratio (95%CI)*

*# Low GII Countries: GII <0.077: Belgium, Denmark, Finland, Netherlands, Norway, Sweden, Slovenia*

*Medium GII Countries: GII: 0.077-0.1635: Austria, Cyprus, Czech Republic, Germany, Greece, France, Spain, Croatia, Ireland, Iceland, Italy, Luxemburg, Poland, Portugal, UK, Lithuania;*

*High GII Countries: GII>0.1635: Bulgaria, Estonia, Hungary, Malta, Romania, Slovakia, Latvia*

*§ Interaction between sex and gender was assessed via repeated sets of multivariable models including two-way interaction between each gender variable and sex.*


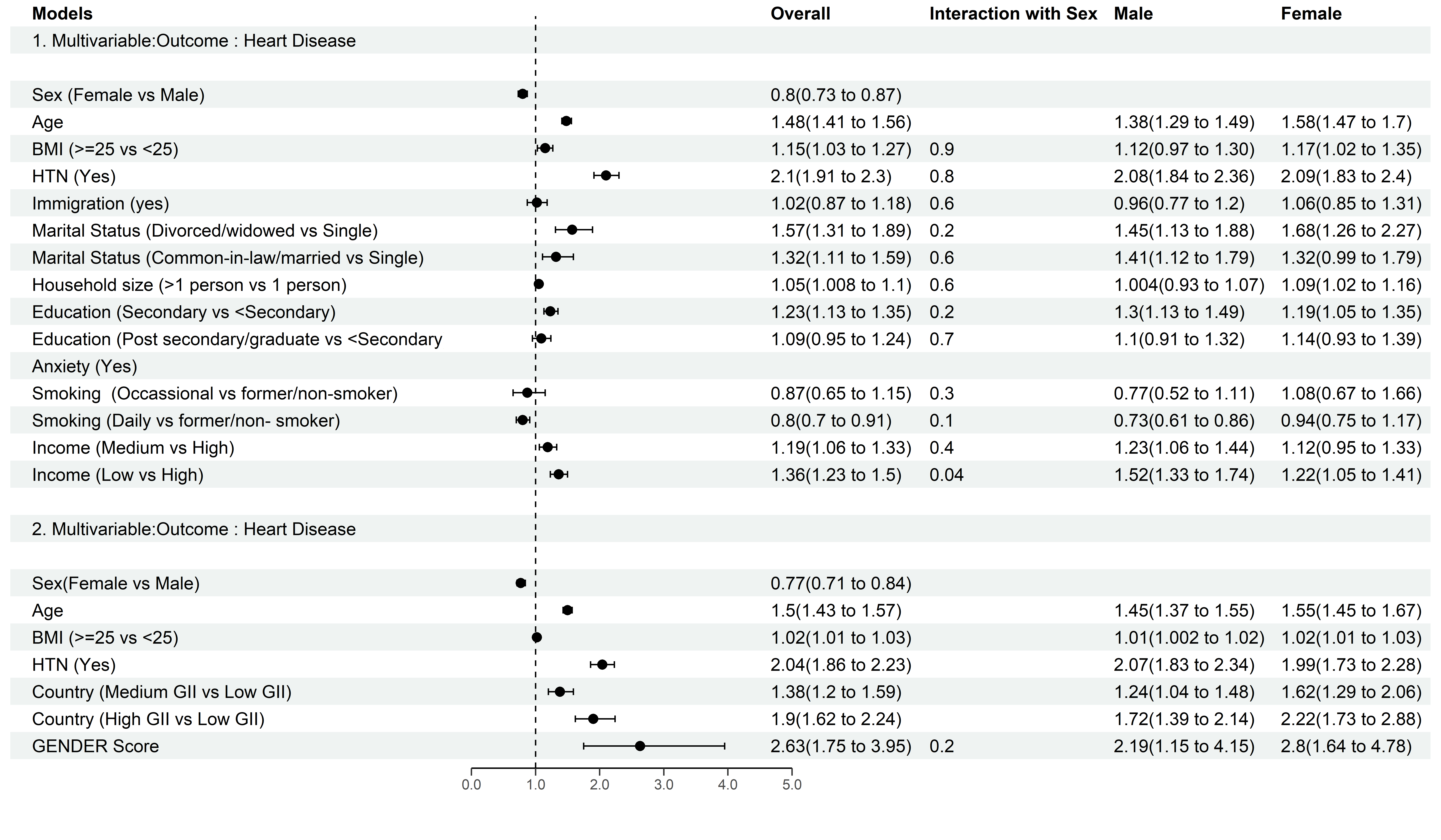


**Appendix16: Forest plot: Role of biological sex and gender variables in predicting stroke of individuals with diabetes in Canadian population:**

**Results are presented as Odds Ratio (95%CI)*

*§ Interaction between sex and gender was assessed via repeated sets of multivariable models including two-way interaction between each gender variable and sex.*


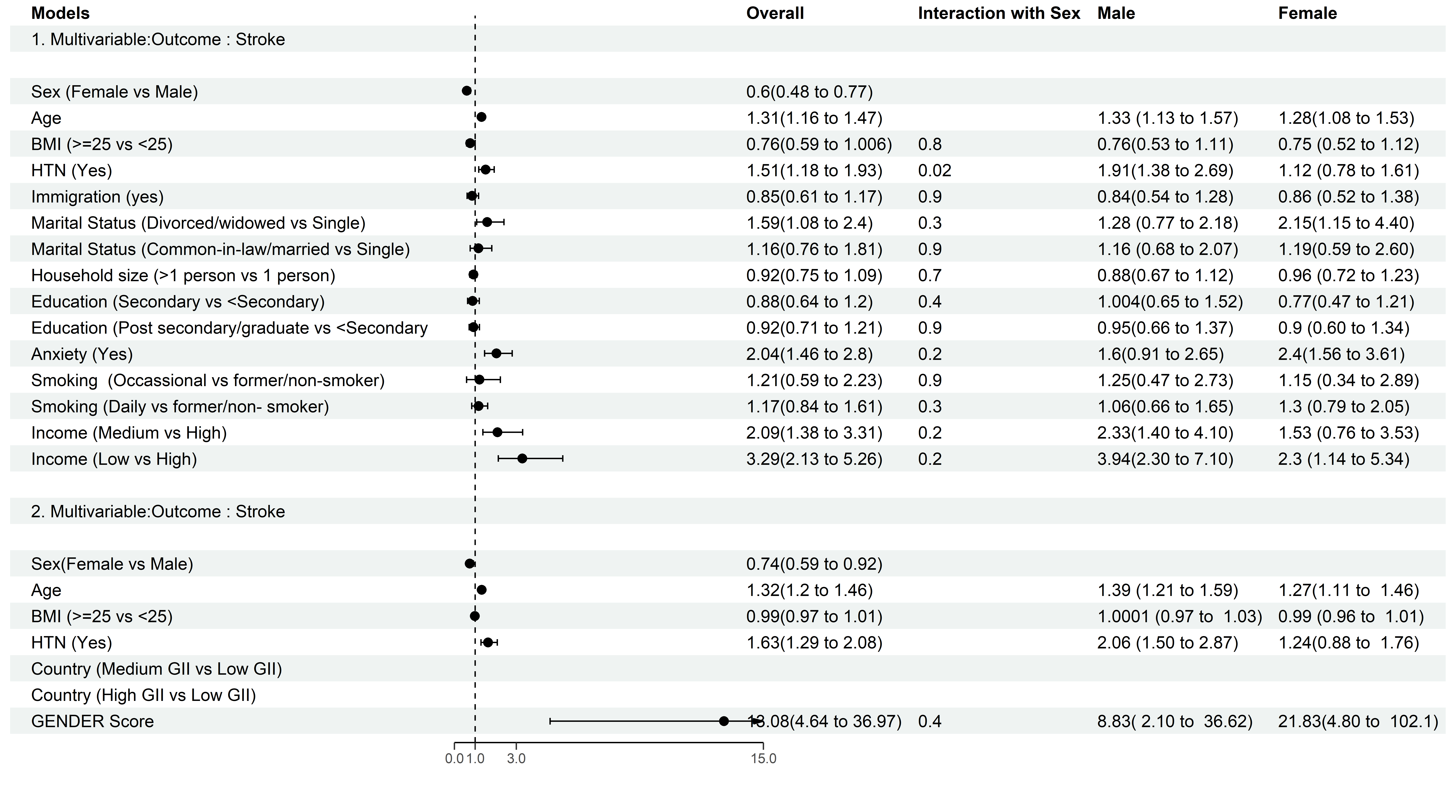


**Appendix17: Forest plot: Role of biological sex and gender variables in predicting stroke of individuals with diabetes in European population:**

**Results are presented as Odds Ratio (95%CI)*

*# Low GII Countries: GII <0.077: Belgium, Denmark, Finland, Netherlands, Norway, Sweden, Slovenia*

*Medium GII Countries: GII: 0.077-0.1635: Austria, Cyprus, Czech Republic, Germany, Greece, France, Spain, Croatia, Ireland, Iceland, Italy, Luxemburg, Poland, Portugal, UK, Lithuania;*

*High GII Countries: GII>0.1635: Bulgaria, Estonia, Hungary, Malta, Romania, Slovakia, Latvia*

*§ Interaction between sex and gender was assessed via repeated sets of multivariable models including two-way interaction between each gender variable and sex.*


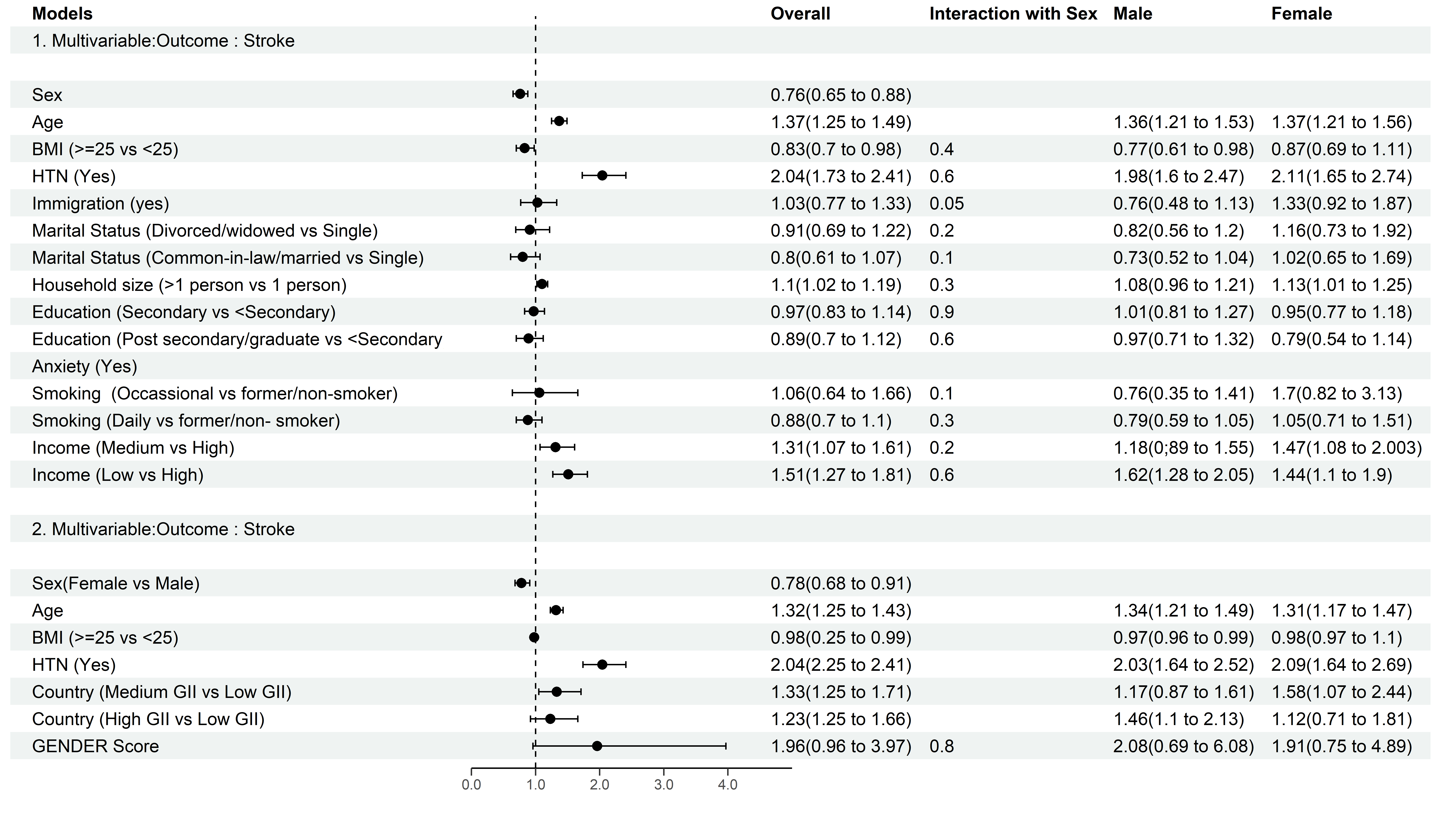


**Appendix18: Forest plot: Assessing role of biological sex and gender variables in hospitalization rate of individuals with diabetes in Canadian population:**

**Results are presented as Odds Ratio (95%CI)*

*§ Interaction between sex and gender was assessed via repeated sets of multivariable models including two-way interaction between each gender variable and sex.*


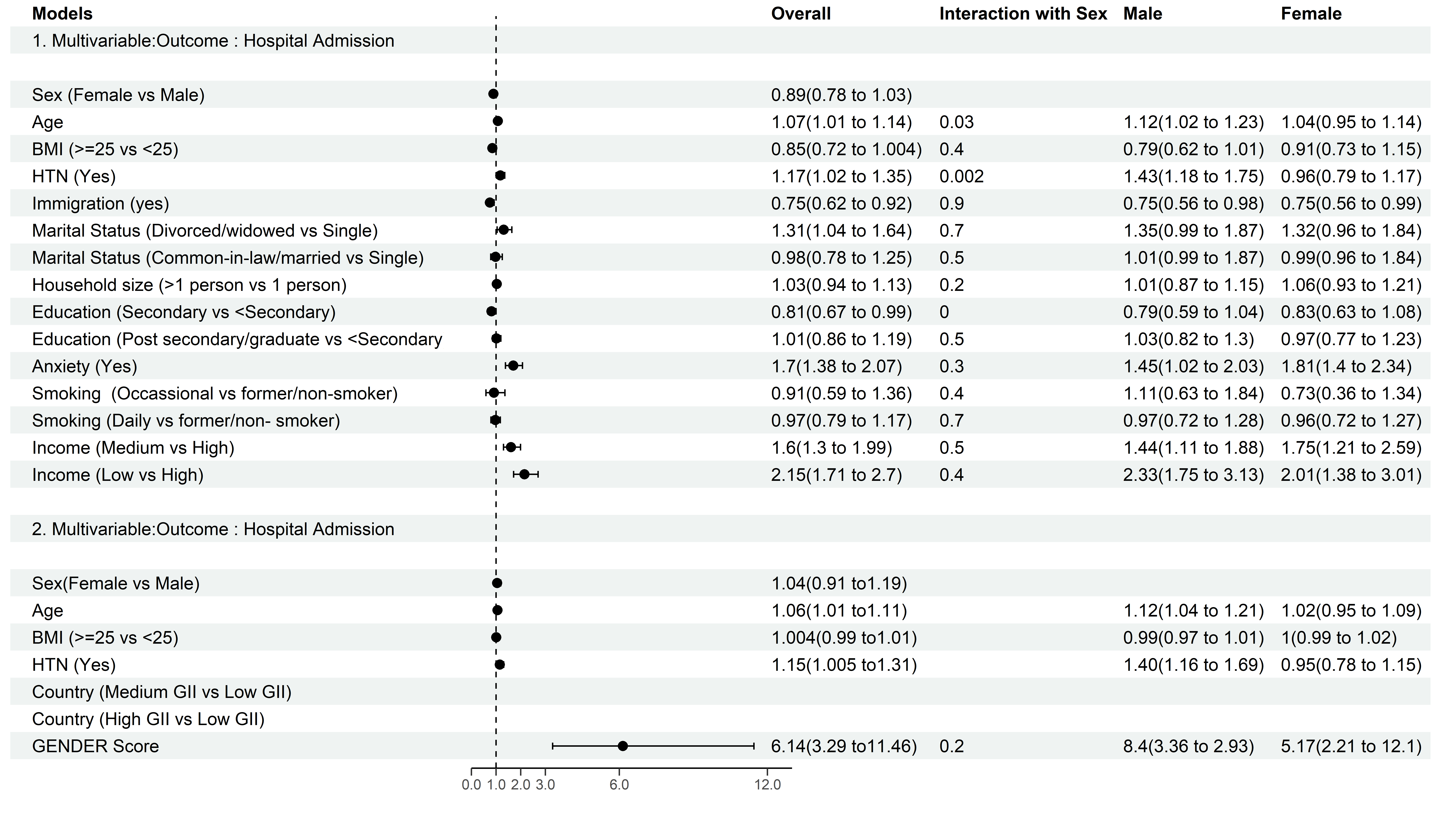


**Appendix19: Forest plot: Assessing role of biological sex and gender variables in hospitalization rate of individuals with diabetes in European population:**

**Results are presented as Odds Ratio (95%CI)*

*# Low GII Countries: GII <0.077: Belgium, Denmark, Finland, Netherlands, Norway, Sweden, Slovenia*

*Medium GII Countries: GII: 0.077-0.1635: Austria, Cyprus, Czech Republic, Germany, Greece, France, Spain, Croatia, Ireland, Iceland, Italy, Luxemburg, Poland, Portugal, UK, Lithuania;*

*High GII Countries: GII>0.1635: Bulgaria, Estonia, Hungary, Malta, Romania, Slovakia, Latvia*

*§ Interaction between sex and gender was assessed via repeated sets of multivariable models including two-way interaction between each gender variable and sex.*


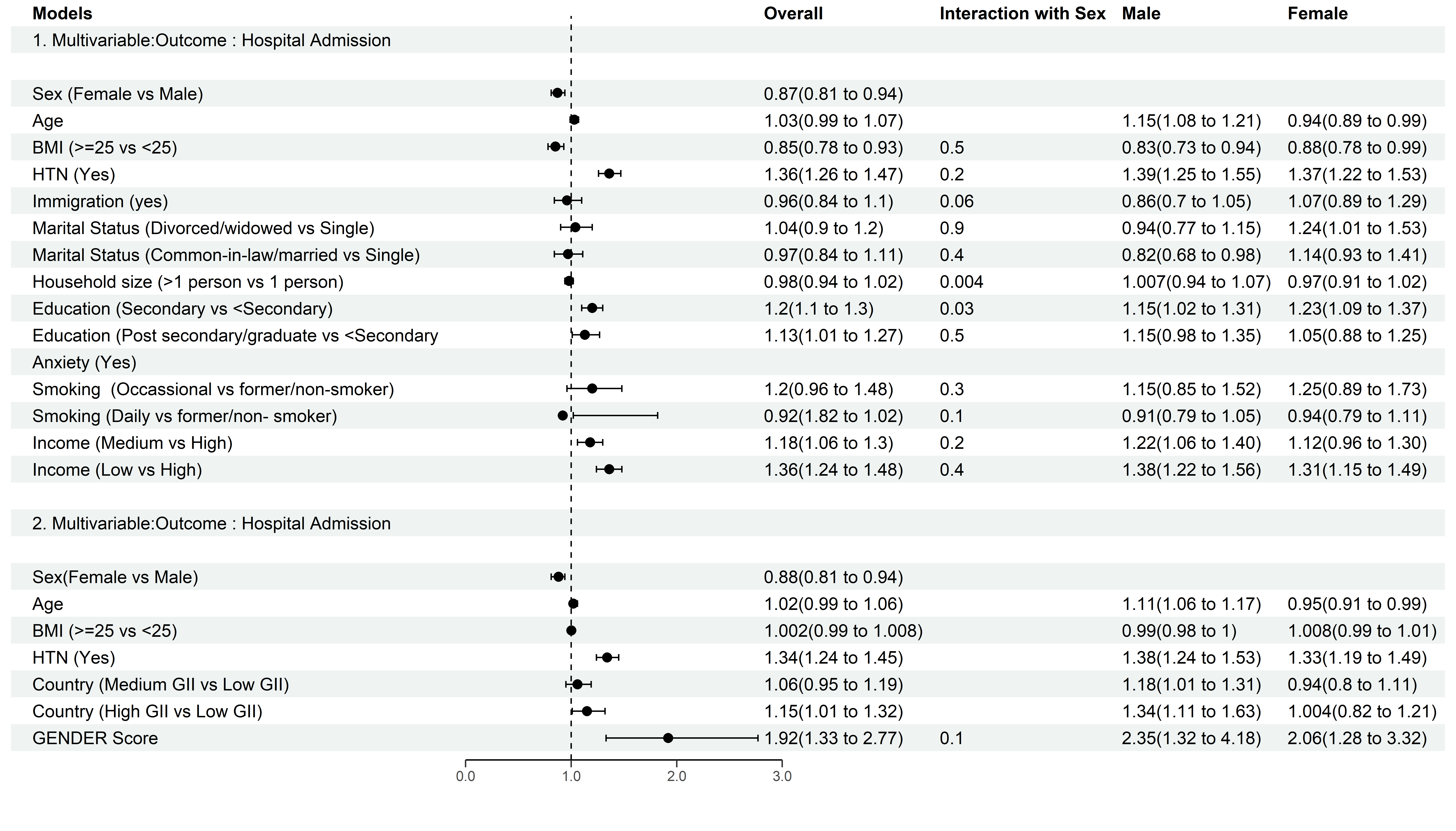


**Appendix 20: Map of Europe. Colors reflect GII. Green=low GII, yellow=middle GII, red=high GII. GII= Gender Inequality Index**
